# Biosensors and Drug Delivery in Oncotheranostics Using Inorganic Synthetic and Biogenic Magnetic Nanoparticles

**DOI:** 10.3390/bios12100789

**Published:** 2022-09-25

**Authors:** Tatiana M. Zimina, Nikita O. Sitkov, Kamil G. Gareev, Viacheslav Fedorov, Denis Grouzdev, Veronika Koziaeva, Huile Gao, Stephanie E. Combs, Maxim Shevtsov

**Affiliations:** 1 Department of Micro and Nanoelectronics, Saint Petersburg Electrotechnical University “LETI”, 197022 Saint Petersburg, Russia; 2 Laboratory of Biomedical Nanotechnologies, Institute of Cytology of the Russian Academy of Sciences, 194064 Saint Petersburg, Russia; 3 SciBear OU, Tartu mnt 67/1-13b, Kesklinna Linnaosa, 10115 Tallinn, Estonia; 4 Research Center of Biotechnology of the Russian Academy of Sciences, Institute of Bioengineering, 119071 Moscow, Russia; 5 Key Laboratory of Drug-Targeting and Drug Delivery System of the Education Ministry, West China School of Pharmacy, Sichuan University, Chengdu 610041, China; 6 Department of Radiation Oncology, Klinikum Rechts der Isar, Technical University of Munich, 81675 Munich, Germany; 7 National Center for Neurosurgery, Nur-Sultan 010000, Kazakhstan

**Keywords:** magnetic nanoparticles, biogenic magnetic nanoparticles, magnetotactic bacteria, magnetosomes, biosensors, drug delivery, oncotheranostics

## Abstract

Magnetic nanocarriers have attracted attention in translational oncology due to their ability to be employed both for tumor diagnostics and therapy. This review summarizes data on applications of synthetic and biogenic magnetic nanoparticles (MNPs) in oncological theranostics and related areas. The basics of both types of MNPs including synthesis approaches, structure, and physicochemical properties are discussed. The properties of synthetic MNPs and biogenic MNPs are compared with regard to their antitumor therapeutic efficiency, diagnostic potential, biocompatibility, and cellular toxicity. The comparative analysis demonstrates that both synthetic and biogenic MNPs could be efficiently used for cancer theranostics, including biosensorics and drug delivery. At the same time, reduced toxicity of biogenic particles was noted, which makes them advantageous for in vivo applications, such as drug delivery, or MRI imaging of tumors. Adaptability to surface modification based on natural biochemical processes is also noted, as well as good compatibility with tumor cells and proliferation in them. Advances in the bionanotechnology field should lead to the implementation of MNPs in clinical trials.

## 1. Introduction

Screening and early diagnostics of oncological diseases are important factors of successful treatment. At the same time, for timely diagnosis, it is necessary to detect minor amounts of markers of such diseases [1]. The modern development of nanotechnologies opens up new opportunities for creating highly sensitive means of diagnostics and treatment of cancer. Currently, such problems are solved by developing new biosensor systems based on various materials and detection principles.

According to the IUPAC definition, a biosensor is “an autonomous complex device capable of obtaining quantitative or semi-quantitative data using a biorecognizing element (bioreceptor) that is in direct spatial contact with a transforming element (transducer)” [2]. In a broad sense, a biosensor is a device that converts a physical or chemical effect on biological objects into a measurable signal. Three main parts can be distinguished in the biosensor structure as follows: (*i*) *biorecognizing element* (e.g., oligonucleotide or peptide aptamers, antibodies, enzymes, proteins, microorganisms, organelles, cell receptors, etc.), which is a material or biomimetic component with a high degree of selectivity to the target analyte; (*ii*) *transducer* (e.g., optical, electrochemical, acoustic, etc.) that converts the signal of interaction between the biorecognizing element and the analyte into a measurable and quantifiable signal (in most cases, an electrical signal); and (*iii*) *data processing system* that analyzes the received signals and visualizes the measurement results conveniently for the operator.

Modern biosensor technology is an example of the convergence of various scientific and technical areas. The use of a variety of nanomaterials (e.g., nanorods, carbon nanotubes, graphene, quantum dots, etc.) is one of the main means of increasing the sensitivity and selectivity of biosensors [3].

**Figure 1:**
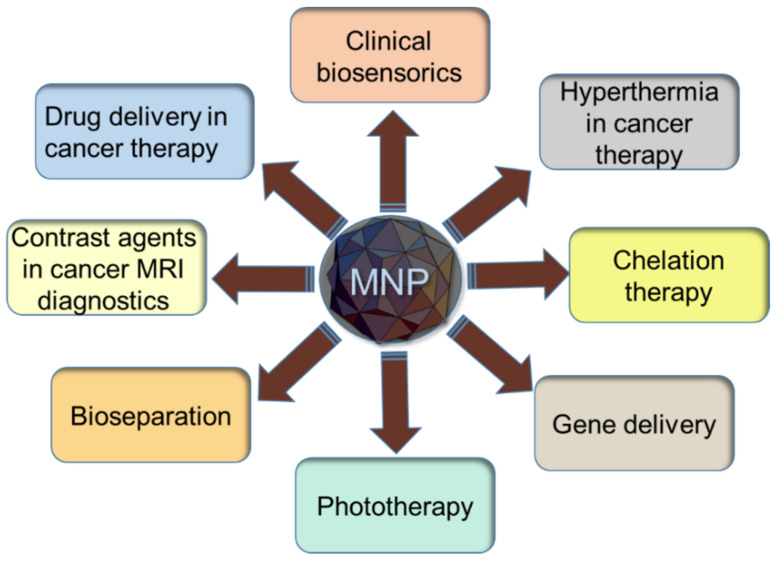
Main clinical applications of magnetic nanoparticles (MNPs).

One rapidly developing direction in the field of theranostics is the utilization of magnetic nanoparticles as transport elements both in sensorics and in therapy by addressing drug delivery. Two main domains of MNPs are used for these purposes: chemically synthesized nanoparticles (which can be synthesized by various methods, such as sol–gel, chemical reduction, co-precipitation, hydrothermal synthesis, and pulsed laser ablation in dimethylformamide and by green methods using plant extracts) [4,5,6,7,8], and biogenic nanoparticles [9,10,11]. There are also other important applications that make magnetic nanoparticles suitable for theranostics (Figure 1).

In recent years, a particular interest is attracted to biogenic magnetic nanoparticles. Since “bacteria with motility directed by the local geomagnetic field have been observed in marine sediments” by Richard Blakemore in 1975 [12], a large number of studies have been carried out in order to determine the properties of such particles, or, so-called magnetosomes, which are the intracytoplasmic membrane vesicles, containing magnetic nanocrystal covered in a lipid bilayer with proteins [13,14,15,16,17,18,19,20]. The most important applications of such biogenic nanoparticles include the biosensors for medical diagnostics, and cancer diagnostics, in particular, vehicles for target delivery of anticancer agents [21], hyperthermia treatment employing alternating electromagnetic field [22], and tumor diagnostics using magnetic resonance imaging (MRI) [23].

In this review, synthetic inorganic and biogenic magnetic nanoparticles will be considered particularly with regard to their applications in biosensors and drug delivery.

## 2. Synthetic Magnetic Nanoparticles

### 2.1. Characterization of Synthetic Magnetic Nanoparticles

Magnetic nanoparticles are biocompatible and lend themselves well to modification by various biorecognition ligands [24]. The main characteristics of MNPs are their subcellular size, ranging from a few nanometers to tens of nanometers, allowing them to interact with nano-molecular-sized biomolecules [25]. Due to their unique properties, magnetic nanoparticles can be used in various biomedical applications including diagnostics [26,27,28], drug delivery [29], hyperthermia treatment [30], tumor cell isolation [31], and precise reagent manipulation [32].

**Figure 2:**
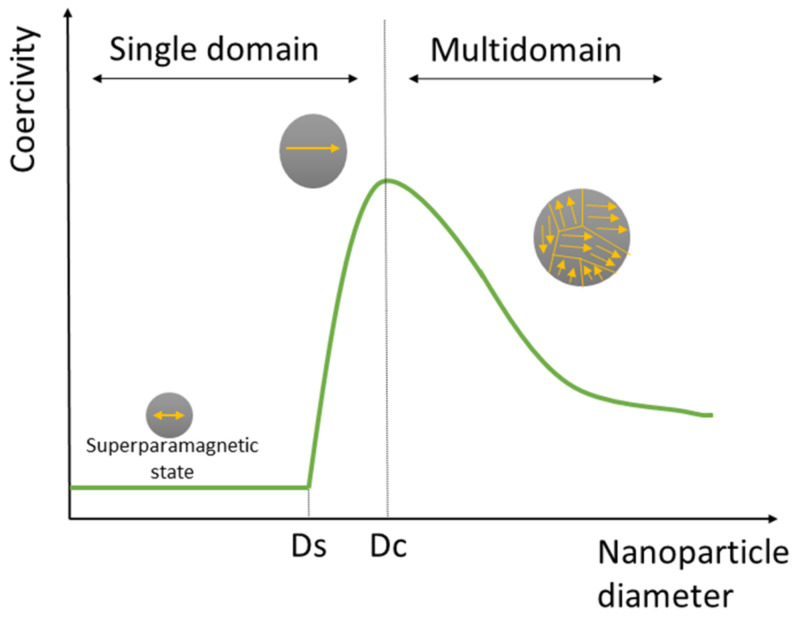
Dependence of coercivity on particle size: Ds and Dc are the thresholds of superparamagnetiс and critical size, respectively.

Ferromagnetic materials are composed of electrically charged particles connected in groups that create magnetic dipoles, which are called magnetons. The volume of a magnetic material is a domain structure in which all magnetons are aligned in a single direction by the exchange forces. This domain structure distinguishes ferromagnetics from paramagnetics [33]. The domain structure of a material is capable of rearrangement as its particle size decreases. This property is one of the main factors determining its ferromagnetic behavior. Figure 2 demonstrates the dependence of the coercive force on the particle size. Reducing the particle size entails an increase in the coercive force up to a certain point—the transition between the single-domain and multi-domain states. The formation of two separate domains below this critical size will be energetically unprofitable. A further decrease in particle size leads to the fact that the coercive force decreases sharply and reaches a minimum in the superparamagnetic state. The transition to the superparamagnetic state induced by thermal effects is not a direct phase transition [34]. MNPs are strongly influenced by an external magnetic field due to the magnetic moment of the network element and the field tension. Therefore, when the external magnetic field disappears, they behave as inactive particles [29]. Synthetic MNPs can take various structural-hierarchical forms depending on the applied magnetic field, such as nanochains, nanorings, nanosheets, and large cuboids [35].

Magnetic nanoparticles based on iron oxides (IONPs), such as maghemite (γ-Fe_2_O_3_), and magnetite (Fe_3_O_4_), are the most common for biomedical applications. Such particles are able to decompose into oxygen and iron and are easily excreted from the organism. When nanoparticles are fabricated of a size of approximately 10 nm in diameter, IONPs exhibit superparamagnetic behavior (superparamagnetic iron oxide nanoparticles, SPIONs), which is due to their better dispersibility without a magnetic field [36]. They accumulate in the target region in the presence of a magnetic field, which is of great importance for use in theranostics. IONPs can be doped with elements with high magnetic susceptibility (e.g., CoFe_2_O_4_, MnFe_2_O_4_, etc.) and metal alloys (e.g., FePt, FeCo, etc.), but their use in living organisms is difficult due to their rapid oxidation and potential cytotoxicity. Multiferroic magnetoelectric (ME) materials such as cobalt ferrite (CoFe_2_O_4_)/barium titanate (BaTiO_3_) nanoparticles have been used for numerous applications, such as biosensors and drug delivery [37,38,39,40]. Their important feature is the presence of a cross-coupling between the electrical and magnetic phases to combine the properties of the individual phases. In ME composites, the application of an external electric field affects the magnetization and vice versa. Crosslinking in composite materials depends on the electrical and magnetic phases, the interface between them, the size, the nature of the connection between the phases, and deformation [41]. One of the most types of common magnetic nanoparticles is «core–shell» structures [42], where the core is a magnetic nanoparticle and its shell is a coating of biocompatible or bioselective material, which allows such structures to be used for drug delivery and in biosensor technology. Biocompatibility and cytotoxicity are key factors to consider when using core–shell MNPs in biomedical applications. Such particles can easily pass through biological membranes and move through the bloodstream, which can lead to disruption of the normal functioning of the systems of a living organism [43]. The presence of a shell on the surface of MNPs is necessary for their stabilization in a colloid system. This will ensure the safety of their interaction with living systems [44]. Hybrid core–shell structures using noble metals such as gold, silver, and platinum are also known [45,46,47]. The optical properties of such MNPs can be precisely tuned by changing the core size, and shell thickness as well as the core and shell shapes [48]. For example, iron oxide–Au MNPs generally exhibit the same magnetic properties as the cores with reduced saturation magnetization due to the mass contribution of the diamagnetic Au and iron oxide–Au core–shell nanostars exhibit multiple plasmonic resonances due to the coupling of the core and tip plasmons [48].

There is a variety of technologies for producing synthetic MNPs that control their size, shape, surface coating, colloidal stability, and other properties, which is especially important for biomedical applications. Magnetic nanoparticles can be manufactured either in a «top-down» or «bottom-up» approach. The «top-down» method involves a high-energy ball milling process of a magnetic sample until the desired nanoscale size is achieved. The advantage of the «top-down» method is that a large number of particles can be produced within a single batch, while the disadvantage is that the control over particle shape and size which is important in biomedical applications, is compromised. The «bottom-up» method starts with a salt of ferrous (Fe^2+^) or ferric (Fe^3+^) ions which then undergo a chemical process to nucleate and induce seeded growth to obtain particles of the desired hydrodynamic diameter [49]. The «bottom-up» approach includes such techniques as hydrothermal, solvothermal, sol–gel, co-precipitation, flow injection syntheses, electrochemical, and laser pyrolysis [50]. Microfluidic methods of synthesizing magnetic nanoparticles, which are based on “lab-on-a-chip” methods and approaches, were also described [51,52]. Such techniques include continuous-flow microreactors and droplet-based microreactors, based on cross-flow, co-flow, and flow-focusing methods, etc. [53,54].

### 2.2. Applications of Biosensors for Cancer Diagnostics

Magnetic nanoparticles (MNPs) are attractive for use in biosensors, since most biological samples have an insignificant magnetic susceptibility, and therefore the background against which measurements are performed is extremely low [55]. MNPs are biocompatible and can be easily modified with various biorecognizing ligands [24].

In biosensorics, the most widely used detection methods, in which MNPs are applied, are optical and electrochemical. Specialized magnetic detection techniques and other techniques are also known. Optical detection methods such as luminescent, fluorescent, colorimetric, etc., are very sensitive and specific. Their principles of operation are based on a change in the phase, amplitude, polarization, or frequency of incoming light in response to biorecognition processes. In such methods, to increase sensitivity, the target molecule or biorecognition element is labeled with a chromogenic or fluorescent label, such as a dye. A change in color/fluorescence intensity indicates the presence of target molecules, which provides high sensitivity with a detection limit of up to one molecule [56]. Electrochemical biosensors use electrodes with recognition elements immobilized on their surface, capable of selectively binding to target molecules. Detection of a target binding to a recognizer in solution is based on the detecting of changes in currents and/or voltages. Electrochemical biosensors (potentiometric, amperometric, and impedimetric) represent detection systems that convert a chemical reaction into a measurable electrical signal. Due to their low cost, low power, and ease of miniaturization, electrochemical biosensors hold great promise for various biomedical applications, especially for Point-of-care Testing (PocT) devices. However, the functioning of these sensors can be influenced by various effects on the electrode surfaces related to pH, ionic strength, and the chemical composition of biological fluids [57]. Magnetic detection methods include various sensing techniques based on giant magnetoresistance, tunneling magnetoresistance, planar Hall effect, etc. These methods are used to measure the magnetic response in the form of susceptibility, relaxation, residual magnetization, and even frequency mixing [58].

Successful cancer treatment remains a challenge where the importance of early tumor diagnostics cannot be overestimated. Therefore, a major goal in modern biosensorics is to increase the sensitivity of detection methods and decrease the analysis time. Magnetic nanoparticles can be easily integrated onto the surface of the transducer or used as a sample preparation component with further concentration of the biological sample in the active region of the biosensor. Due to the growing interest in increasing sensitivity and selectivity, the optimization of MNPs for specific applications and the choice of optimal detection methods are important challenges for modern nanoscience [58]. MNPs can greatly increase the sensitivity of biosensor devices. MNPs exhibit different magnetic properties compared to the bulk material due to the reduced number of magnetic domains, which leads to the appearance of a superparamagnetic state. In this state, the magnetization can randomly change direction in a very short time. This superparamagnetic behavior prevents attractive or repulsive forces between magnetic nanoparticles until an external magnetic field is applied [59].

To diagnose oncological diseases, biosensors based on magnetic nanoparticles use various techniques for modifying them in order to bind highly specific biorecognizing agents, which should capture extremely low analyte concentrations. The variety of detection techniques and biorecognition interfaces used for the recognition of cancer cells are presented in Table 1. Some examples of applications of biosensor devices based on various detection methods using magnetic nanoparticles for cancer diagnosis will be discussed below.

Electrochemical biosensors have become widespread due to a wide range of detection techniques, ease of technological implementation, availability of measuring equipment, and wide potential for miniaturization and integration into lab-on-a-chip systems. The current level of micro- and nanotechnologies has made it possible to create a variety of components of biosensor systems using magnetic nanoparticles, thus providing increasingly lower detectable concentrations of target substances, which is extremely important in cancer diagnostics.

The use of noble metal coatings has become a common technique for creating biorecognizing structures based on magnetic nanoparticles for electrochemical biosensors [60]. Thus, Chen et al. reported the development of an electrochemical biosensor for the detection of DNA methylation in blood-based on square wave voltammetry [60]. This process involves hybridization in a probe network of DNA-modified gold-coated magnetic nanoparticles (DNA-Au@MNP) complementary to the target DNA and subsequent enzymatic digestion to differentiate between methylated and unmethylated DNA strands. The detection limit for the developed biosensor was 2 aM. Another electrochemical sensing assay in combination with the DNA-Au@MNPs was used for the direct detection of the levels of circulating tumor DNA from whole blood [61]. This biosensor can selectively detect short- and long-strand DNA targets with a dynamic range from 2 aM to 20 nM for 22 nucleotide targets and from 200 aM to 20 nM for 101 nucleotide targets, respectively. In another study biosensor based on hollow hybrid magnetic Pt–Fe_3_O_4_@C nanospheres for sarcosine detection was presented by Yang et al. [65]. In order to achieve excellent electron transfer, polyaniline was used as a coating of Pt-Fe_3_O_4_ nanoparticles, which were then pyrolyzed to carbon.

**Figure 3:**
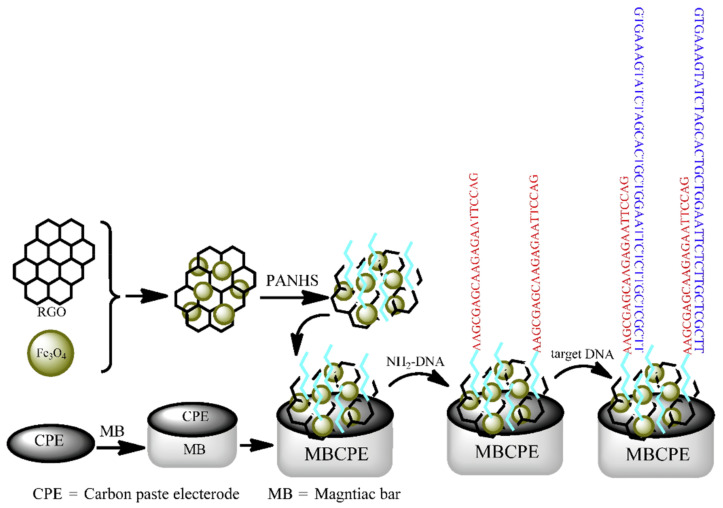
Schematic representation of the modified electrochemical biosensor based on MBCPE/Fe_3_O_4_-RGO/PANHS platform. Reprinted from [66] with permission of Elsevier provided by Copyright Clearance Center.

A promising approach for the implementation of high-sensitivity electrochemical biosensors is the use of magnetic nanoparticles combined with carbon nanomaterials. The biosensor for prostate-specific antigen (PSA) detection was developed based on modification of the glassy carbon electrode (GCE) surface with a nanocomposite of carboxyl functionalized multi-walled carbon nanotubes (MWCNTs), and Fe_3_O_4_ nanoparticles for signal amplification and facilitating electron transfer [62]. The biosensor demonstrated the detection limit of 0.39 pg·mL^−1^ to measure PSA with a linear range from 2.5 pg·mL^−1^ to 100 ng·mL^−1^. Khoshfetrat and Mehrgardi [63] reported an aptamer-based electrochemical biosensor with carbon-modified electrodes for quantitative detection of leukemia cells using MNPs. A composition of Fe_3_O_4_ nanoparticles coated with gold nanoparticles was used to immobilize the thiolated sgc8c aptamer on the surface. The binding of tumor cells to the aptamer leads to the destruction of its hairpin structure. As a result, intercalator molecules are released (ethidium bromide was used for this purpose), which leads to a decrease in the electrochemical signal. Amplification of the signal of the electrochemical platform was provided by the immobilization of nitrogen-doped graphene nanosheets on the electrode surface. Jahanbani et al. [66] designed a label-free DNA biosensor for breast cancer mutation detection (BRCA1 5382 insC) based on a magnetic bar carbon paste electrode (MBCPE) modified with Fe_3_O_4_/reduced graphene oxide (Fe_3_O_4_NP-RGO) as a composite and 1-pyrenebutyric acid-N-hydroxysuccinimide ester (PANHS) as a linker for DNA sequences detection (Figure 3). The MBCPE/Fe_3_O_4_-RGO/PANHS electrode was modified with probe strands for the exact incubation time. This biosensor showed a detection limit of 2.8 × 10^−19^ mol·L^−1^ in a linear range from 1.0 × 10^−18^ mol·L^−1^ to 1.0 × 10^−8^ mol·L^−1^.

MNPs with a “core–shell” structure are among the most frequently used in biosensor applications. At the same time, new variants of shells are being created, which make it possible to detect various cancer cells with high sensitivity. Thus, surface-active maghemite nanoparticles (SAMNs) with a self-assembled coating of Cr^VI^ were used to make a nanostructure (SAMN@Cr^VI^) with immobilized bovine serum amine oxidase (BSAO) [70]. The use of chromate made it possible to bind BSAO, which does not spontaneously bind to the SAMN surface, and the electrochemical signal of the SAMNs was radically changed on the formation of the self-assembled Cr^VI^ shell. The obtained nanoconjugate was employed for interference-free polyamine determination in liver cancer tissues. Wang et al. provided an electrochemical immunoassay method for the detection of α-1-fetoprotein (AFP) based on core–shell–shell nanoparticles functionalized with antibodies [69]. The basic CoFe_2_O_4_/(3-mercaptopropyl) trimethoxysilane nanostructure (CoFe_2_O_4_–MPS) was synthesized by covalent conjugation. Then, gold nanoparticles were sorbed onto the surface of this nanostructure using the Au–S bond, and then anti-AFP antibodies were immobilized. MNPs doped with biomolecules were attached to the electrode surface by applying an external magnetic field. The voltammetric biosensor was performed in a linear range from 0.8 to 120 ng·mL^−1^ AFP concentration range with a detection limit of 0.3 ng·mL^−1^.

Optical detection systems are also widely used in biosensors due to their high sensitivity. Nevertheless, in this group of methods, it is also important to provide signal amplification for the detection of ultra-low analyte concentrations. Therefore, magnetic nanoparticles are also used in this group of methods.

**Figure 4:**
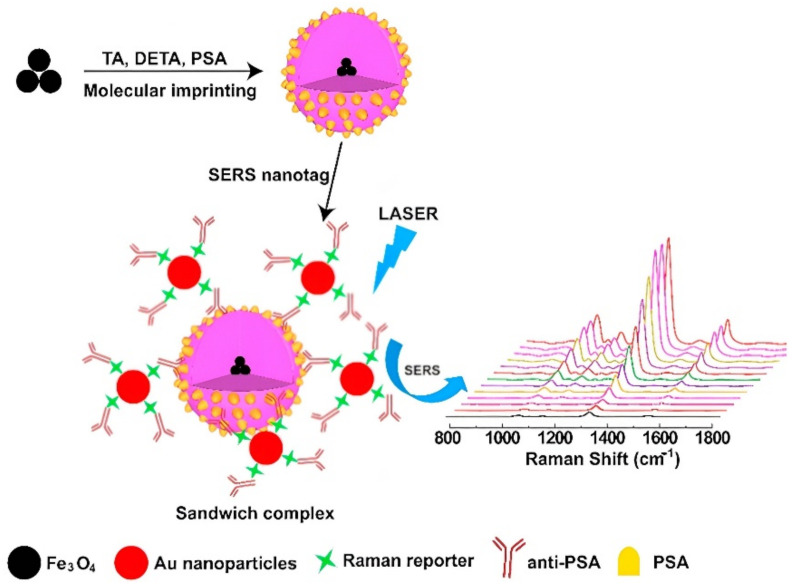
The schematic of plasmonic biosensor for prostate-specific antigen by combining magnetic molecularly imprinted polymer and surface-enhanced Raman spectroscopy. Reprinted from [72] with permission of Elsevier provided by Copyright Clearance Center.

A common optical detection method is surface-enhanced Raman scattering (SERS). This method offers the creation of quite low-cost multisensor systems, which is especially important for the development of Point-of-care Testing devices. Pang et al. [71] reported a surface-enhanced Raman scattering (SERS) detection system of the total RNA extracted from tumor cells using a hybrid Fe_3_O_4_@Ag MNPs biosensor functionalized with DNA probes. A single target miRNA molecule can rehybridize thousands of DNA probes to trigger signal-enhancing recycling in the presence of an endonuclease duplex-specific nuclease (DSN). The superparamagnetic properties of Fe_3_O_4_@Ag hybrid MNPs allowed capturing, concentration, and direct quantification of target miRNA let-7b without any PCR preamplification treatment. Additionally, MNPs may be used as a structural component of molecularly imprinted polymers (MMIPs) for SERS biosensors [72]. This sandwich structure served as an antibody-free probe and was labeled with gold nanoparticles that were modified with anti-PSA and a Raman reporter. This allowed to create a plasmonic structure between the MMIP and the SERS label. The detection and quantification limits of the developed sensor were 0.9 pg·mL^−1^ and 3.2 pg·mL^−1^, respectively (Figure 4). 

Multiparametric surface plasmon resonance can be effectively used as a label-free technique for studying the process of dynamic mass transfer in the nanoparticle/cell system in the fluid cell. Shipunova et al. [73] obtained a spectrum of colloidally stable MNPs modified with phytolectins (SBA, WGA, ConA) of different specificity for monosaccharides (GalNAc, GlcNAc, and Man, respectively) and studied the interaction of these conjugates with A431 human epidermoid carcinoma cells. The authors showed that not only the angle of the minimum peak in the full angular spectrum but also the intensity of this peak can be used to study the binding of target MNPs to living cells in dynamics. This is explained by the contribution of metal nanoparticles to the absorption of incident electromagnetic radiation by free electrons by the resonance mechanism at the interface between media with different refractive indices. The combination of label-free SPR and magnetometric MPQ cytometry techniques allowed to establish that MNPs modified with soybean agglutinin bind to epidermoid carcinoma cells reaching saturation in 12 min to 4.2 ± 0.1 pg·cell^−1^ [73].

**Figure 5:**
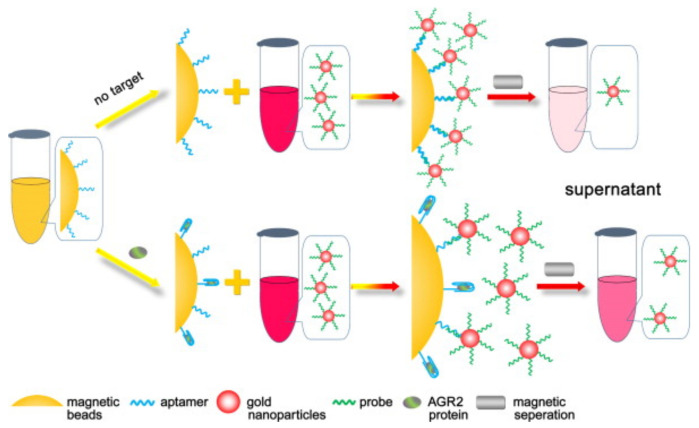
Schematic representation of AGR2 protein detection procedure. Reprinted from [75] with permission of Elsevier provided by Copyright Clearance Center.

Spectrometric techniques can also be used for high-sensitive cancer biomarkers detection. One example of such a system is the work [75]. Here, the principle of UV–visible spectrometry was used to develop a sandwich-type aptasensor based on gold nanoparticles/DNA/magnetic beads to detect the cancer biomarker protein AGR2. The obtained structure (Figure 5), built on the high affinity between the aptamer and the target protein and providing a picomolar detection limit, made it possible to determine the target analyte with a sample volume of up to 20 µL. It was also noted that the sensitivity and selectivity of the developed sensor can be improved using magnetic separation.

**Figure 6:**
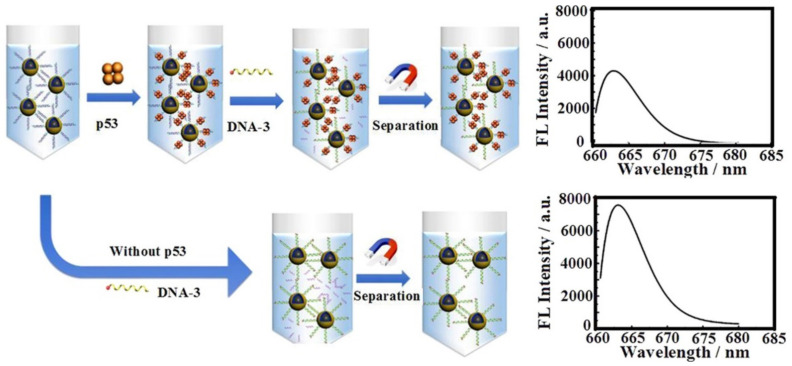
A fluorescent sensor for p53 protein expression was developed by combination of functional consensus DNA and magnetic nanoparticles. The sensor can realize ultrasensitive detection of p53 protein in real cell lysate. Reprinted from [76] with permission of Elsevier provided by Copyright Clearance Center.

Another example of spectrometric detection in biosensor systems is fluorescence registration. A fluorescent biosensor for p53 protein quantification was explored using DNA/dextran/PAA/Fe_3_O_4_ nanocarriers by Xu et al. [76]. Dextran-aminated MNPs were used to functionalize the consensus DNA that can selectively bind wild-type p53 protein (Figure 6). Dextran coating reduced nonspecific protein absorption and the sensitivity for p53 protein was achieved due to the facile magnetic separation from the complex condition. Inhibition of the process of DNA replacement by the captured p53 protein on the DNA consensus domain provided a decrease in fluorescent emission. Another promising approach for cancer detection war presented by Lee et al. [77], who developed cancer-cell-derived exosomes biosensor via the magnetofluoro-immunosensing (MFI) system using hybrid Ag/iron oxide NP-decorated graphene (Ag/IO-GRP) without purification and concentration processes. The authors successfully detected a prostate-cancer-cell-derived exosome from non-purified exosomes in a culture media sample in a concentration range from 10^2^ NPs·mL^−1^ to 10^6^ NPs·mL^−1^.

Colorimetric methods of registration are based on measuring the optical density at a given wavelength, thus making it possible to determine the concentration of the substance in question. The result of the analysis can be recorded quite easily with a mobile device. A fast colorimetric immunosensor was developed on the basis of a nanocomposite of platinum and magnetic nanoparticles incorporated into mesoporous carbon [79]. This system allowed highly specific detection of human epidermal growth factor receptor 2 (HER2) at room temperature within 3 min. The technology exemplifies a strategy, in which nanocomposites are utilized for rapid, robust, and convenient identification of target pathogens. Consequently, the approach has potential applications for point-of-care (PoC) detection in clinical diagnostics.

**Figure 7:**
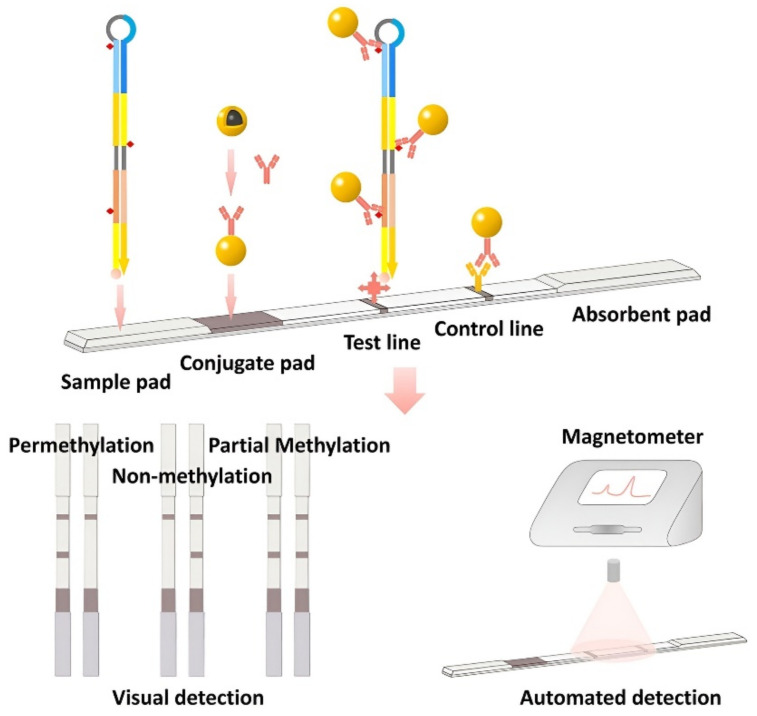
The schematic of DNA methylation biosensor by combination of isothermal amplification and lateral flow device. Reprinted from [80] with permission of Elsevier provided by Copyright Clearance Center.

The combination of Immunochromatographic and magnetometric techniques is quite promising for use in biosensors. The combination of loop-mediated isothermal amplification (LAMP) and lateral flow device (LFD) was used to identify traces of DNA methylation from highly heterogeneous cancerous specimens [80]. Gold magnetic nanoparticle (GMNP), working as a signal generator in this biosensor, enabled to interpret DNA methylation patterns through both visual and magnetic representation. The result can be obtained through both visual and magnetic detection. The performance of this biosensor was verified with real-world samples in the determination of the DNA methylation pattern of miR-34a promoter (Figure 7). Another site-specific biosensor based on lateral flow assay was established for both visual and magnetic DNA methylation determination [81]. The introduction of primers labeled with digoxin and biotin into PCR made it possible to recognize amplicons that can be recognized and captured by gold magnetic nanoparticles (GMNPs) using the developed biosensor device. The optical properties of GMNPs make it possible to use them as a signal probe and interpret amplicons even with the naked eye. The magnetic properties of these particles make it possible to register a signal using a magnetometer. The combination of such detection techniques is promising for use in clinical practice.

**Figure 8:**
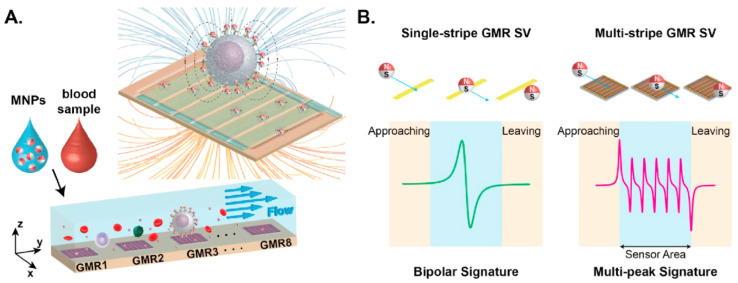
Magnetic flow cytometer (MFC) concept: (**A**). Operation of a GMR SV-based MFC where MNP decorated cells flow over GMR SV sensors. (**B**). Signature from conventional single-stripe sensors with a simple bipolar peak that increases the false detection events and the proposed multi-stripe configuration that enhances the signal differentiation by creating a unique magnetic signature. Reprinted from [82] with permission of Elsevier provided by Copyright Clearance Center.

The unique magnetic properties have also found their application in biosensors for cancer diagnostics. One of these properties is giant magnetoresistance, devices based on which can be integrated into miniature analytical systems. Magnetic flow cytometers are a possible solution for rapid cancer cellular detection in PoC testing devices. Thus, Huang et al. [82] reported an array of microfluidic biosensors based on a giant magnetoresistive spin valve with multiband sensor geometry and matched filtering to improve detection accuracy without sacrificing throughput (Figure 8). When cells labeled with MNPs pass by, the sensor generates a characteristic signal, which allows measurements to be taken in a multiparametric mode. The throughput of the developed device for multiparametric measurements was 37–2730 cells/min.

Another magnetoresistive biosensor based on an InSb channel was demonstrated by Kim et al. [83]. The Fe_3_O_4_ nanoparticles bound to the target antigen created a stray magnetic field, which induced a change in semiconductor channel resistance due to the Lorentz force. The antigen concentration was proportional to the number of MNPs attached to the sensor surface and, therefore, could be determined by measuring the magnetoresistance of the sensor channel. Zhu et al. presented a GMR biosensor for exosome detection based on aptamer-modified 2D MoS_2_-Fe_3_O_4_ nanostructures providing both multidentate targeting and signal amplification [85]. Unlike pure MNPs, layered MoS_2_ nanostructure acts as a recruitment matrix for high-density MNPs as magnetic probes. The developed GMR sensor using 2D magnetic nanocomposites demonstrated reproducibility and selectivity with a detection limit of 100 exosomes.

Multiferroic magnetoelectric nanoparticles (MENs) are an attractive tool for the development of new magnetic tools for cancer diagnostics. For this, Nagecetti et al. [84] used such 30-nm core–shell particles as probes synthesized by the solvothermal method. In such a system, the electric and magnetic fields are inextricably linked. Due to the clear association with cells and the magnetoelectric effect, the NMR absorption spectra for cells incubated with MENs differed significantly from cells without such particles. Ordinary MNPs caused only minor changes in the adsorption spectra or did not cause them at all. The authors concluded that the minimization of the magnetoelectric energy upon binding of nanoparticles to cells is responsible for the change in the NMR adsorption spectrum in the case of MEN.

Thus, the variety of implementations of biosensor systems using MNPs for cancer diagnostics clearly indicates broad prospects for the introduction of these systems into clinical practice. Undoubtedly, the key task in creating such systems is to increase their sensitivity, stability, reproducibility, reliability, as well as the economic availability of testing. Nevertheless, the creation of multiparametric biosensor systems for cancer diagnostics remains an important task, since large-format POCT screening makes it possible to form a map of human health and exclude many causes of diseases at once. This will allow us to formulate the optimal strategy for the treatment or preventive correction of the patient’s condition.

### 2.3. Synthetic Magnetic Nanoparticles for Drug Delivery

Following diagnostics, modern technologies should provide conditions for the successful treatment of oncological diseases. Most anticancer drugs (e.g., doxorubicin, paclitaxel, curcumin, etc.) are administered intravenously, which often leads to a significant number of side effects [86,87,88,89,90,91]. The major limitation of present chemotherapeutic agents is the poor selectivity and the resultant toxicity [92]. Therefore, to minimize the negative effects of anticancer drugs, various systems are being developed for their targeted delivery directly to tumor cells [93].

**Figure 9:**
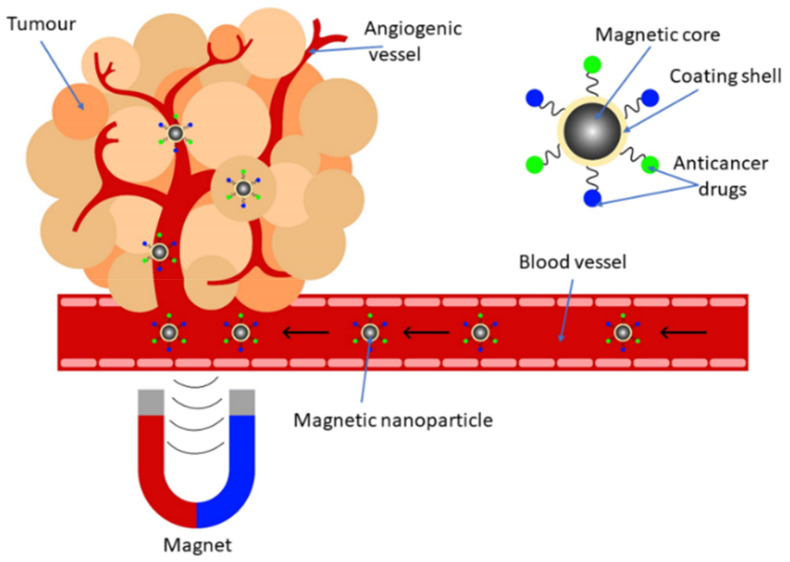
Scheme of targeted drug delivery using magnetic nanoparticles.

Magnetic nanoparticles have become one of the most developing means of targeted drug delivery against cancer due to the possibility of their modification with various shells that improve their biocompatibility, allowing them to attach more active substances to them and avoid aggregation in the blood vessels. The size of MNPs is an extremely important factor for their therapeutic use since particles with a diameter of less than 10 nm are quite easily excreted through the renal clearance, while those larger than 200 nm are absorbed by the spleen [94]. To bring MNPs with an anticancer drug to the site of the tumor, high-gradient rare-earth metal magnets are mainly used, which make it possible to focus the magnetic field in the desired area. However, as the distance of the tumor from the body surface increases, the effectiveness of such targeting decreases, since the strength of the applied magnetic field decreases [95]. Figure 9 shows a scheme for targeted delivery of anticancer drugs using MNPs.

Since toxicity remains the main challenge in the use of magnetic nanoparticles in anticancer drug delivery, to overcome this, new methods of modifying MNPs into biocompatible shells with chemotherapeutic agents attached to them are being developed. In addition, it is necessary to overcome the agglomeration and aggregation of MNPs, which can also cause negative consequences for the body and complicate the complete release of drugs in the tumor site.

The variety of anticancer drugs necessitates the creation of an optimal delivery system for each of them to the tumor site in order to provide the most effective treatment. Oncotheranostics and nanomedicine are currently developing quite intensively and are now becoming one of the key areas in the creation of new methods of cancer treatment. Therefore, we propose to consider in this part of the review only fairly recent examples of the use of magnetic nanoparticles for targeted drug delivery. Table 2 shows the structures of systems for targeted delivery of various anticancer drugs based on the work of the last 5 years.

As can be seen from Table 2, a very wide range of coatings for magnetic nanoparticles is used to deliver various anticancer drugs. The variety of coatings used to create targeted drug delivery systems based on magnetic nanoparticles suggests that the optimal composition of such a coating has not yet been found, which would minimize the toxicity of the system and make it fully biocompatible. Below we consider some examples of such structures with different coatings.

**Figure 10:**
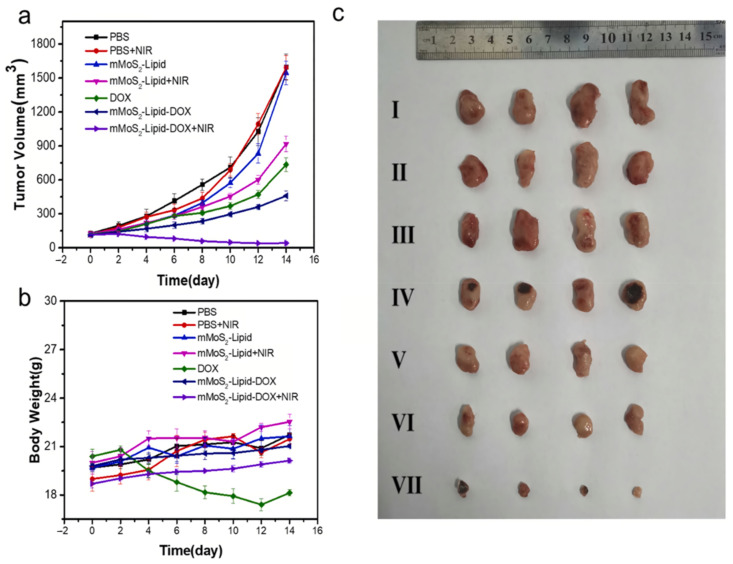
The changing curve of tumor volume from the beginning to the end of treatment (mean ± SD, *n*  =  4) (**a**). The weight change curve of mice in each group (mean ± SD, *n*  =  4) (**b**). The tumor image of different groups of mice on the 14th day following the treatment (mean ± SD, *n*  =  4) (**c**). Reprinted from [106] with permission of Elsevier provided by Copyright Clearance Center.

One of the most common coatings for drug delivery using MNPs is liposomes. A nanocomposite based on Fe_3_O_4_ nanoparticles coated with liposomes loaded with bufalin was presented as a drug for inhibiting lymphatic metastasis in breast cancer [97]. MNPs performed a targeting and photothermal function, accumulating in the sentinel lymph nodes of laboratory mice. The proposed technique allowed to reduce the incidence of lung metastases by 81% and achieve 91% tumor inhibition in the sentinel lymph nodes of mice. Lee et al. reported on doxorubicin nanocarriers based on liposome-coated magnetic molybdenum disulfide (mMoS_2_) used for combined photochemotherapy [106]. The nanocarriers demonstrated a rather low rate of nonspecific protein adsorption and a low degree of aggregation in physiological saline. A reasonably successful cellular uptake profile of MCF-7 cells without significant cytotoxicity was obtained from in vitro studies. While in vivo studies (Figure 10) demonstrated that a drug delivery system based on mMoS_2_ and liposomes provides tumor inhibition in mice with fewer negative effects.

**Figure 11:**
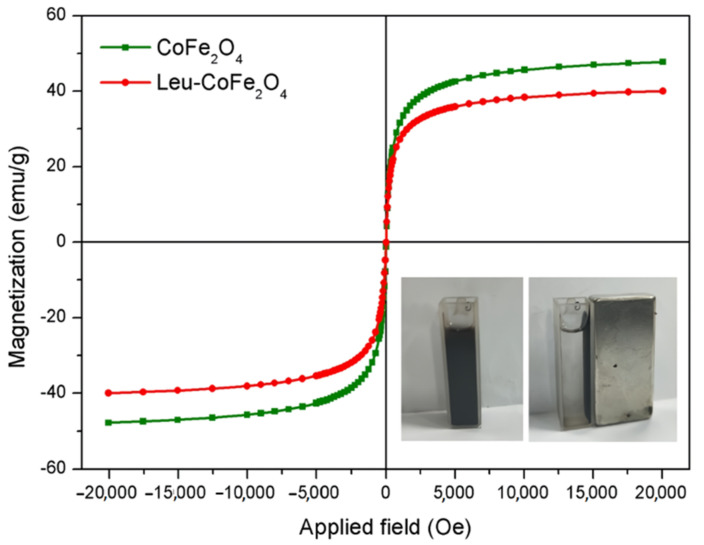
Magnetization curves of bare CoFe_2_O_4_ and Leu-coated CoFe_2_O_4_ nanoparticles. The inset shows the process of dispersion and magnetic separation. Reprinted from [105] with permission of Elsevier provided by Copyright Clearance Center.

Amino acids are also one of the widely used coatings for magnetic nanoparticles in oncotheranostics. Thus, L-cysteine-encapsulated ZnFe_2_O_4_ nanoparticles in combination with oxygen-containing functional groups and a nitrogen-rich mesoporous graphite phase with carbon nitride were used as a biodegradable target sonodynamic chemotherapeutic agent for tumor eradication [100]. The developed nanocomposite served as a carrier of the anticancer drug curcumin with a pH and ultrasound trigger, as well as to perform a semi-enzymatic sonocatalytic function. In another study, Attari et al. [90] proposed a method for the preparation of iron oxide MNPs coated with arginine using the in situ co-precipitation method and the one-pot method. The obtained nanoparticles were covalently bound to methotrexate and can target most cancer cells whose surface is overexpressed with folate receptors. Due to the functionalization of nanoparticles with arginine, an amide bond appeared on their surface between the amino groups and terminal carboxylic acid grappa on methotrexate, which was released from the nanoparticles in the presence of protease-like lysosomal conditions. Experiments on cell lines MCF-7, 4T1, and HFF-2 demonstrated the absence of cytotoxicity, which makes the developed system promising for use in clinical practice. In [105], solvothermally synthesized nanoparticles of cobalt ferrite (CoFe_2_O_4_) coated with leucine were used as a doxorubicin delivery system. The developed nanocarriers not only showed the ability to effectively inhibit the proliferation of HeLa cells, exerting an obvious cytotoxic effect on them but also demonstrated high sensitivity to a magnetic field in comparison with CoFe_2_O_4_ nanoparticles without leucine coating (Figure 11).

**Figure 12:**
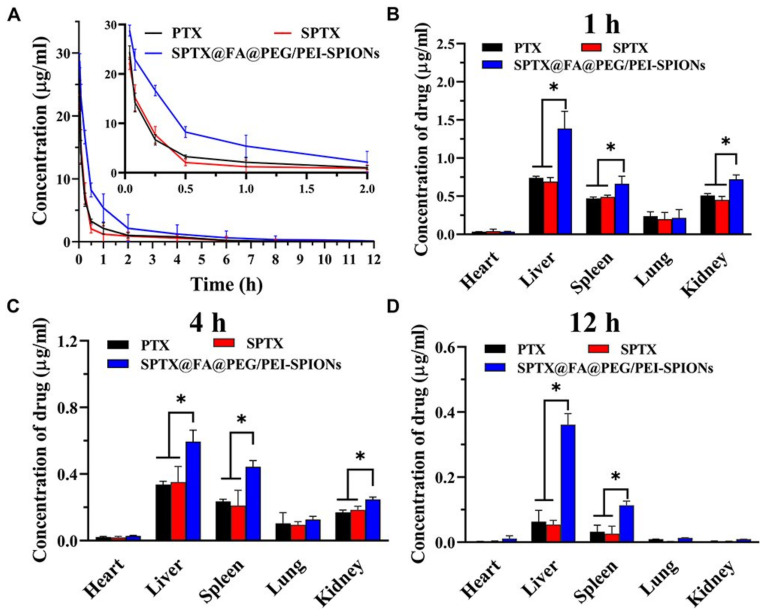
Plasma concentration–time curves of free PTX, SPTX, and SPTX@FA@PEG/PEI-SPIONs in vivo, data are expressed as the mean ± SD (*n* = 4) (**A**). Tissue distributions of free PTX, SPTX and SPTX@FA@PEG/PEI-SPIONs at 1 h (**B**), 4 h (**C**) and 12 h (**D**) post-intravenous injection (*n* = 4), * *p* < 0.05. Reprinted from [88], license CC BY 3.0.

Another promising direction in the development of magnetotherapeutic preparations is the use of polymer compositions as coatings for magnetic nanoparticles. Jin et al. [113] proposed polyethyleneimine-coated Fe_3_O_4_ MNPs for delivery of therapeutic siRNAs targeting B-cell lymphoma-2 and Baculoviral IAP repeat-containing 5 into Ca9-22 oral cancer cells. The study demonstrated significant inhibition of Ca9-22 cell viability and migration as a result of the use of nanoparticle-delivered siRNAs. Noh et al. [103] obtained a nanocomplex responsive to the tumor intracellular microenvironment, co-assembled from a copolymer of polyethylene glycol and poly (aspartic acid), superparamagnetic iron oxide nanoparticles (SPIONs), and doxorubicin. The performance of this therapeutic nanocomplex was studied on cell lines of colon carcinoma and fibroblasts. Moreover, NPs showed enzymatic degradation in the presence of protease, as well as a contrast effect on magnetic resonance imaging. Gui et al. presented a composite nanosystem based on folic acid (FA)-loaded SPIONs designed to reduce adverse reactions to water-insoluble parcitaxel (PTX) [88]. An increase in the hydrophilicity of PTX was achieved by modifying it with succinic anhydride, thus obtaining’-succinate parcitaxel (SPTX). SPTX-loaded FA-conjugated polyethylene glycol (PEG)/polyethyleneimine (PEI)-SPION (SPTX@FA@PEG/PEI-SPION) was prepared by solvent volatilization and hydrogen bond adsorption. Pharmacokinetic studies in rats in vivo on nasopharyngeal carcinoma cells (Figure 12) showed that SPTX@FA@PEG/PEI-SPIONs particles had a longer duration of action (t_1/2_ = 3.41 h) than free SPTX or PTX (t_1/2_ = 1.67 h).

**Figure 13:**
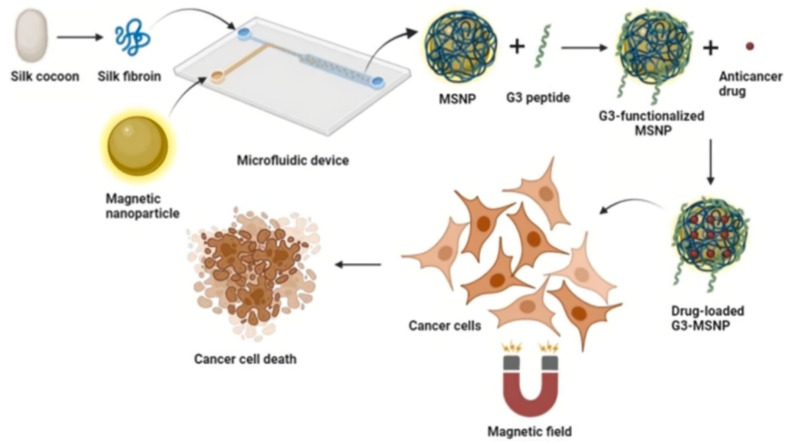
A schematic of peptide-functionalized magnetic silk nanoparticles produced by a swirl mixer for enhanced anticancer activity of ASC-J9. Reprinted from [111] with permission of Elsevier provided by Copyright Clearance Center.

To reduce toxicity and increase biocompatibility, magnetic nanoparticles can be coated with proteins. Chen et al. used high-voltage electrospray technology to develop microspheres based on Fe_3_O_4_ nanoparticles with a gelatin shell for loading them with adriamycin [96]. In addition to a good antitumor efficacy, the obtained nanocomplex activated ferroptosis in tumor cells (the ferroptosis marker GPX4 was significantly decreased, and ACSL4 was significantly increased) together with exposure to microwave hyperthermia, and also showed excellent properties for magnetic resonance imaging. Another interesting application of protein coatings is work [90], where bovine serum albumin-coated MNPs were used as curcumin carriers. Nanospheres prepared through desolvation and chemical co-precipitation process demonstrated cytotoxic activity on the MCF-7 cell line and sustained release of curcumin at 37 °C in different buffer solutions. Tomeh et al. [111] developed a microfluidic method for the production of peptide-functionalized magnetic silk nanoparticles based on silk fibroin for targeted delivery of the hydrophobic anticancer agent ASC-J9 (Figure 13). A swirl mixer integrated into a microfluidic chip allowed to achieve the required shape and size for the synthesized MNPs. Their surface was functionalized with a cationic amphiphilic antitumor peptide G (IIKK)_3_I-NH_2_ (G3) in order to increase the selectivity to cancer cells. The resulting complex increased the anticancer activity and cellular uptake of the G3 peptide in HCT 116 colorectal cancer cells as compared to the free G3 peptide.

The use of polysaccharides as coatings for MNPs is another common option for creating nanosystems for targeted drug delivery based on MNPs. Dextran is widely used for this purpose. In the study [98], SPIONs coated with dextran and conjugated with folic acid were synthesized by co-precipitation to deliver camptothecin to prostate cancer cells. The nanocarriers, which were spherical with an average diameter of 63.31 nm, demonstrated antitumor activity in AT3B-1 cancer cells by actively releasing and delivering camptothecin at 37 °C in phosphate and citrate buffers. The MNPs presented in [102] coated with polyarabic acid (a water-soluble polysaccharide molecule) and loaded with doxorubicin demonstrated effective penetration through cell membranes and internalization into breast cancer cells in a mouse model. The developed nanomaterials have demonstrated good biocompatibility, low cytotoxicity in vitro and in vivo, as well as the possibility of using them as a contrast agent in MRI.

**Figure 14:**
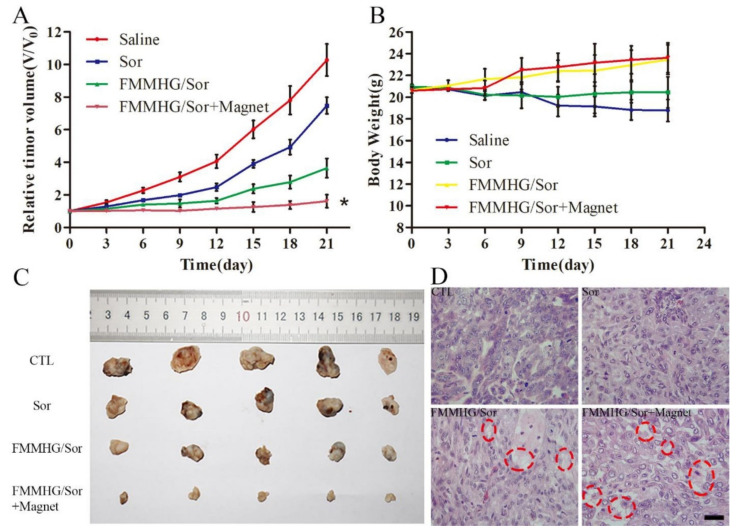
In vivo antitumor therapeutic effect of FMMHG/Sor. (**A**) The tumor volume of mice after being treated with saline, free Sor, FMMHG/Sor, and FMMHG/Sor + magnet for 21 days. * *p* < 0.05. (**B**) The body weight of mice in each group (saline, free Sor, FMMHG, and FMMHG + magnet) with treatment for 21 days. (**C**) Representative photos of tumor tissues obtained in different groups (saline, free Sor, FMMHG, and FMMHG + magnet) after 21 days of treatment. (**D**) Histological section of tumor tissues with H&E staining of different groups (saline, free Sor, FMMHG, and FMMHG + magnet) after 21 days of treatment. The fat vacuoles were marked with a red circle, which was the sign of ferroptosis. Scale bars, 100 μm. Reprinted from [114] with permission of Elsevier provided by Copyright Clearance Center.

It is also of interest to form non-toxic coatings on magnetic nanoparticles based on mesoporous materials. Recently, Abolhasani Zadeh et al. [104] proposed mesoporous hematite MNPs loaded with doxorubicin as a multifunctional theranostic agent exhibiting therapeutic activity against human MCF-7 breast cancer cells. These biomimetic mesoporous MNPs have over 71% doxorubicin loading efficiency resulting in a 50% reduction in cancer cells at a concentration of 0.5 µg·ml^−1^. The obtained MNPs, having a polygonal structure with an increased surface area and high porosity, became suitable nanocontainers for a high loading of doxorubicin. Another promising method for effective tumor cell killing is ferroptosis, which bypasses apoptosis and overcomes tumor drug resistance. Thus, acid- and redox-sensitive MNPs loaded with sorafenib developed by Chi et al. effectively stimulated tumor ferroptosis and inhibited tumor growth in vivo [114] (Figure 14). Mesoporous organosilicon nanoparticles (MONs) were coated on the outside with Fe_3_O_4_ MNPs, which provided sufficient iron ions for ferroptosis and magnetic targeting. As a result, a core–shell nanostructure was formed, which contained a disulfide bond with a redox reaction. MnO_2_ was dropped onto the surface of the MON as a pylorus, which degraded to O_2_ at low pH to promote sorafenib release. Hyaluronic acid acted as a protector of the nanoparticles from removal by the immune system and promoted active targeting of cancer cells. Another interesting study demonstrated the use of mesoporous magnetic MnFe_2_O_4_ core–shell nanocomposite particles for poorly water-soluble quercetin delivery [116]. MnFe_2_O_4_ nanoparticles as a core of nanostructure were made with the co-precipitation method. Then, the synthesized MNPs were coated with mesoporous hydroxyapatite (HA) shell as a new perspective for drug loading. The magnetic mesoporous nanostructure had a specific surface area and mean pore size of 165.44 m^2^/g and 11.561 nm, respectively, which provided the possibility to efficiently load QC into the MNPs’ pores with the subsequent pH-dependent release of the agent.

Multiferroic magnetoelectric nanoparticles are also used in the targeted delivery of anticancer drugs. Stewart et al. [110] studied the externally controlled anticancer effects of binding synthetic tumor growth-inhibiting peptides to CoFe2O4@BaTiO3 magnetoelectric nanoparticles (MENs) in the treatment of glioblastomas. MIA-class growth hormone-releasing hormone antagonist molecules (MIA690) were chemically linked to these particles and then tested in vitro on human glioblastoma (U-87MG) cells. Studies have demonstrated externally controlled, highly efficient binding of MIA690 to MEN, specificity for glioblastoma cells, and on-demand release of the peptide using d.c. and a.с. magnetic fields, respectively. The work [108] presents colloidally stable MENs of cobalt ferrite @ barium titanate (CoFe_2_O_4_@BaTiO_3_) synthesized by the sonochemical method and further functionalized with doxorubicin and methotrexate. In vitro cytotoxicity studies performed on hepatocellular carcinoma (HepG2) and human malignant melanoma (HT144) cells confirmed the magnetoelectric properties of CoFe_2_O_4_@BaTiO_3_ NPs in the presence of an external magnetic field (5 mT) with significantly increased cytotoxicity compared to free preparations and without field replicates.

Metal–organic frameworks (MOFs) are becoming a promising tool for drug delivery applications. Pandit et al. [115] reported the development and fabrication of a dual MOF composite with encapsulated iron oxide (IO) nanoparticles coated with folic acid (FA) as the targeting agent and quercetin (Q) as the drug agent. Due to the presence of SPION, composites inherently show potential use for MRI. The integration of dual zeolite imidazolate frameworks (ZIF-8/ZIF-67) with targeting agents and drugs demonstrated the effective anticancer activity of the obtained nanocomposites (IO/Z8-Z67/FA/Q) in an FA receptor-positive breast cancer cell culture model (MDA-MB-231). The resulting nanocomposite enhanced apoptosis and cytotoxicity in the MDA-MB-231 cell line (expressing folate receptors) compared to the MCF-7 cell line, in which folate receptors were absent. Mechanically, the folic acid receptor targeting the delivery of the IO/Z8-Z67/FA/Q nanocomposite to MDA-MB-231 cells caused high ROS generation and nuclear fragmentation, which led to cell death. The proposed nanocomposite was also used for 5-fluorouracil loading, and the results of cytotoxicity suggest that it is a versatile nanocarrier for targeted drug delivery.

Hybrid structures containing nanoparticles of iron oxide and noble metals have also found application in the targeted delivery of anticancer drugs. The system prepared by Liu et al. [107], was based on dextrin-coated silver NPs, which were then cross-linked with iron oxide NPs and a cell-penetrating peptide (Tat), resulting in dual-functional Tat-FeAgNPs with both superparamagnetic and cell-penetrating properties. The resulting nanocomplex can first overcome the blood flow shear force and reach the target organ under the action of an external magnetic field, and then the surface-modified Tat can further promote tissue penetration, which can effectively improve the efficiency of targeted drug delivery. The results showed that the obtained nanocomplex can promote cellular uptake and cytotoxicity of nanoparticles loaded with doxorubicin, while the IC_50_ of Tat-FeAgNP-Dox was 0.63 µmol·L^−1^. Nie et al. [121] fabricated platinum (Pt) nanozymes dispersed on the surface of iron oxide (Fe_3_O_4_) nanospheres loaded with 5-fluorouracil (FLU), which, in addition to enhancing peroxidase-mimic activity and catalsimic activity, led to the formation of a pH-sensitive nanoplatform for drug delivery for breast cancer treatment. Cytotoxicity tests showed that the obtained Fe_3_O_4_/Pt-FLU@PEG nanospheres moderate the proliferation of 4T1 cancer cells mediated by apoptosis and intracellular production of reactive oxygen species. In vivo assays have shown a significant reduction in tumor size and overcoming tumor hypoxia.

Since MNPs are foreign objects to the body, therefore, the immune system rejects them and various toxic reactions are caused. Therefore, the targeted delivery of drugs to the human body requires the control of such parameters of MNPs as shape, size, homogeneity, and coating composition. The coating plays an essential role in various applications: being highly biocompatible and stable, it would allow the adhered biomolecules to remain active for longer within the body and satisfactorily control their release or remaining attached for long periods to be stored and used for diagnosis [50]. MNPs synthesis and formulations face critical biological barriers, such as localization at the target site, the effective delivery of the drug to the target site, cross-physiological talk, and the other technical obstacles specific to cancer [122]. The development of precision drug delivery systems based on magnetic nanoparticles will make it possible to implement highly effective oncotheranostic techniques and improve the quality of life of patients.

## 3. Biogenic Nanoparticles

### 3.1. Biogenic Synthesis and Diversity of Magnetic Nanoparticles

Biogenic synthesis of nanoparticles can be carried out by organisms such as bacteria [123,124,125,126], fungi [127], lichens [128], and algae [129]. The production of biogenic nanoparticles is environmentally friendly since the synthesis process takes place at ambient temperature and pressure, and no toxic chemicals are used [130]. Hence, many researchers are focusing on synthesizing biogenic nanoparticles over chemically or physically synthesized ones to produce inexpensive, energy-efficient, and non-toxic metal nanoparticles [131]. Various types of naturally synthesized metallic nanoparticles consist mainly of Ce, Ag, Au, Pt, Pd, Cu, Ni, Se, Fe, or their oxides [132]. Among them, a particular interest is attracted to magnetic nanoparticles [133].

**Figure 15:**
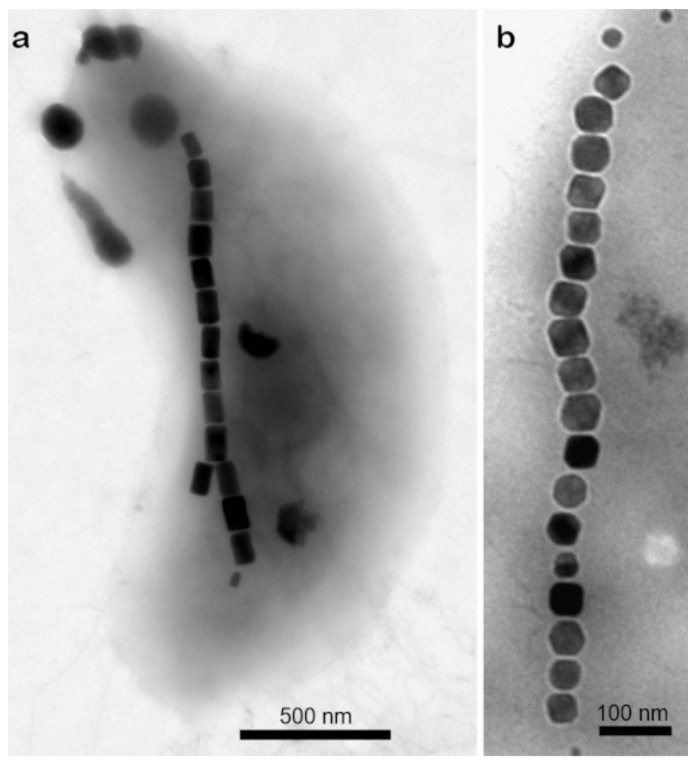
TEM image of (**а**) MTB with a chain of prismatic magnetosomes, (**b**) a chain of magnetosomes with a visible membrane.

Magnetotactic bacteria (MTB) synthesize magnetosome magnetic particles with a well-controlled size and morphology, covered with an organic membrane 3–4 nm thick, which provides high and uniform, compared to artificial magnetite, dispersion in aqueous media, making them ideal biotechnological materials [134] (Figure 15).

**Figure 16:**
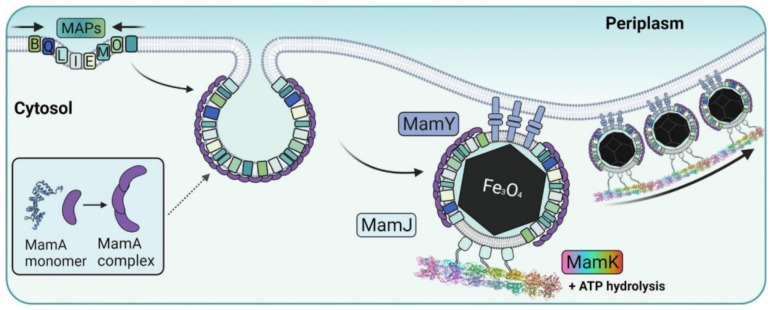
Suggested model of protein sorting, membrane invagination, and magnetosome assembly into an organized chain. Proteins solved structures are in ribbon representation. Reprinted from [137], license CC BY-NC-ND 4.0.

Based on the results obtained in a detailed study of *Magnetospirillum gryphiswaldense* MSR-1 and *M. magneticum* AMB-1 strains, the mechanism of magnetosome biomineralization was suggested, with *mam* genes being mainly involved. Magnetosome formation is a complex process that has been divided into 4 steps, each of which involves certain Mam proteins [135,136,137,138]. The magnetosome membrane is the result of the invagination of the cytoplasmic membrane [139,140]. The initiation point of the invagination process is apparently not determined by the specific composition of lipids in the membrane but rather is triggered by the presence of certain Mam proteins on it [137]. The minimal protein complex MamLQBIEMO enables proper invagination, whereas magnetite biomineralization requires additional proteins [119]. Among them, the membrane protein MamB is probably the most crucial [135,136]. The sorting stage inlolves the addressing of magnetosome proteins to the forming vesicle. Presumably, MamA protein plays an important role in this process, since it is present on the magnetosome membrane surface in large quantities [141,142] (Figure 16). MamA contains a repetitive protein–protein interaction site, which provides its oligomerization and the ability to bind other proteins [143]. Since MamA completely covers the magnetosome membrane, it can serve as a receptor for other magnetosome proteins [141,144]. For example, MamC, which is one of the most abundant proteins on magnetosome membranes in wild-type cells, was found to be mislocalized in *Magnetospirillum gryphiswaldense* MSR-1 mutants with MamA deletion [145].

After the creation of the magnetosome vesicle, the transport of the corresponding ions in and out of the vesicle takes place to synthesize magnetite or greigite in the magnetosomes [146]. Such proteins as MamB and MamM, two carriers of divalent iron cations from the cell into the magnetosome, are involved [147]. When optimal physicochemical conditions are achieved in the magnetosome vesicle, one magnetite crystal per magnetosome is synthesized, which has a species-specific morphology [148]. Alignment into the chains occurs simultaneously with nucleation and crystal growth [137]. MamK, MamJ, and MamY are taking part in the chain organization [139,149].

The ability to form magnetosomes has been found in many bacteria from more than 10 prokaryotic phyla with different physiology [150,151,152,153]. However, all isolated in axenic culture MTB or those that have morphological descriptions and genomic sequences belong to the phyla *Pseudomonadota (classes* *Alphaproteobacteria*, *Gammaproteobacteria*, and *Magnetococcia),* *Thermodesulfobacteriota Nitrospirota*, and *Omnitrophota* (Table 3). Most MTB have magnetosomes organized into one or more chains. On average, MTB contains several tens of magnetic particles, and some species, such as *Candidatus* Magnetobacterium bavaricum, contain up to 1000 magnetosomes per cell [154]. The magnetic core crystals of magnetotactic bacteria are of different shapes which depends on MTB species. The majority of MTB can synthesize only one type of crystal, either magnetite or greigite. BW-1, however, was shown to be able to synthesize both types of crystals, depending on the sulfide concentrations in the medium [155]. The crystal size, crystallographic orientation, and arrangement of the magnetosomes in the MTB are crucial for the magnetic properties of the cell [156].

The magnetosomes extracted from MTB meet all requirements in terms of size, morphology, biocompatibility, and magnetization capability [210,211,212,213,214]. However, despite the wide diversity of MTB and the remarkable properties of magnetosomes, only a few species from the phylum *Pseudomonadota* are cultivated and used to study the mechanisms of magnetosome formation and their applications [215]. Considerable efforts have been devoted to the production and purification of magnetosomes to obtain large yields of stable magnetic nanoparticles [216]. For example, studies have been carried out to optimize growth conditions [217,218] and transfer magnetosome genes into fast-growing non-magnetotactic strains [219].

Separation of MTB with different magnetic contents with subsequent isolation and enrichment can be performed using microfluidic devices based on their magnetic contents [220]. The motility of MTB can overcome magnetic forces, causing false positives (reduced purity) and false negatives (reduced yield). To overcome the movement of bacteria, MTB strains were treated with a cold/alkaline medium (10 °C, pH 8.5). Magnetosome production and growth were unaffected by this treatment. Thus, high-throughput separation of *Magnetospirillum gryphiswaldense* MSR-1 (1.000 cells/μL × 25 μL/min = 25,000 cells/min) was achieved with up to 80% sensitivity and 95% isolation purity. This demonstrates that microfluidic technology can greatly facilitate the separation of MTB cells with the required magnetic properties.

Isolating intact magnetosome organelles is an essential technique used in biotechnological applications. Magnetosomes from disrupted cells can be purified by means of their magnetic attraction with a permanent magnet and further ultracentrifugation in a sucrose density gradient [142,221,222]. However, despite extensive washing magnetically enriched magnetosomes still contained numerous contaminating proteins from other cellular fractions [223]. The use of nano and microfluidic technologies can be adapted for the study and isolation of magnetosomes and MTB [220,223,224].

### 3.2. Applications of Magnetosomes in Cancer Theranostics

#### 3.2.1. Biosensors on the Basis of Magnetosomes

Magnetosomes (MS), due to uniform size and morphology (Figure 15), highly ordered organic membrane (Figure 16), and ability to form homogeneous dispersions offer a perspective substrate in biotechnological applications, particularly biosensors [134]. Thus, their advantages over inorganic magnetite nanoparticles, include, first of all, a stable single-domain form of the magnetic core which provides a permanent magnetic state at ambient temperature, then—a high chemical purity, a narrow size distribution, etc. These properties enable MS to be applied for cell identification and isolation on-a-chip, antigen detection and recovery, enzyme immobilization and capture of target proteins, and contrast enhancement in magnetic resonance imaging (MRI) [224,225,226].

In [227], the authors described an original method of toxicity detection, in which the magnetic properties of magnetosomes within the magnetotactic bacteria are combined with bioluminescence ability. These features are achieved by genetic engineering on the basis of magnetotactic bacteria *Magnetospirillum gryphiswaldense,* strain MSR-1. The approach made it possible to obtain a hybrid organism (BL-MTB) that combines the magnetic properties of navigation with the ability to emit a red glow of click beetle luciferase, and the latter property turned out to be proportional to the viability of the bacterium [227]. The magnetic navigation ability of bacterium served as a “natural actuato” to provide transport of bacteria within a microfluidic chip from the reactor to the detection volume. As a result, a cost-effective biosensor for toxicity monitoring was developed using microfluidic technology implemented on a polydimethylsiloxane (PDMS) molded chip. This analytical technology is quite express, since BL-MTB are incubated for 30 min with the sample, moved by microfluidics, trapped, and concentrated in detection chambers by an array of neodymium–iron–boron magnets [227].

A biosensor for white spot syndrome virus (WSSV) detection was presented in [228]. The biosensor is implemented on the basis of antigen-antibody reaction of VP28-specific antibodies conjugated with magnetosomes at concentrations of 1 and 2 mg·mL^−1^ and VP28 antigen at concentrations of 0.025~10 ng·µL^−1^. The complex was transported to carbon planar electrodes in a magnetic field applied externally and the antigen concentration was determined using an electrical impedance measurement principle. The assay was applied in monitoring seafood samples contaminated with WSSV and VP28 antigen of concentration as low as 0.01 ng·µL^−1^ was detected. Thus, magnetosomes were successfully applied in biosensors for detecting viruses, due to the possibility of biorecognition ligands conjugation to their native surface and the capability of addressing positioning and concentration of target particles at detection areas due to magnetic carriers [228].

**Figure 17:**
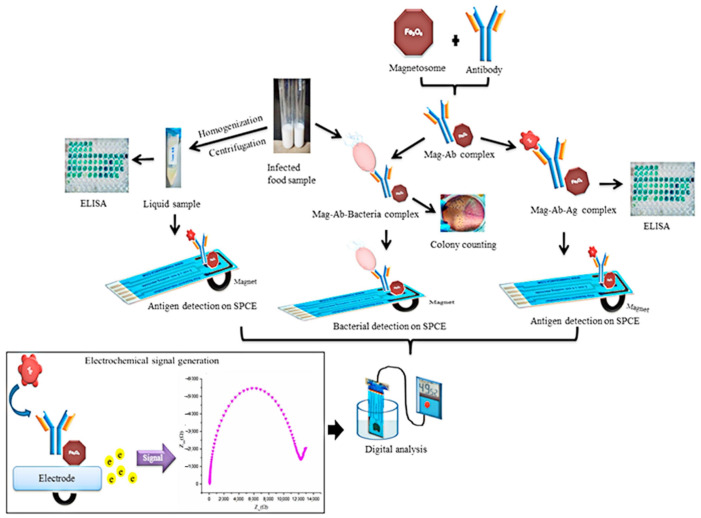
Schematic illustration of lipopolysaccharide and *Salmonella typhimurium* detection using magnetosome-anti-Salmonella antibody complex. Reprinted from [229] with permission of Elsevier provided by Copyright Clearance Center.

A similar detection principle was implemented in biosensors for the determination of pathogenic bacteria and their toxins [229]. The authors presented a biosensor of *Salmonella typhimurium* employing a magnetosome with immobilized antibodies for the “O” antigen of *Salmonella* lipopolysaccharide (Figure 17). The optimal MS-Ab complex concentrations for detection of lipopolysaccharide concentration of 1 ng·mL^−1^, were in the range of 2 mg·mL^−1^~0.8 μg·mL^−1^. An external magnet was used for the concentration of the probe at the area of the electrode. The reaction was detected using the electrical impedance principle. In real samples, the biosensor demonstrated high sensitivity with the bacteria detection limit of 101 CFU·mL^−1^ [229]. Figure 17 shows schematically the advantages of the developed biosensor, which integrates on a single chip a number of operations applied in conventional analysis of pathogenic organisms.

An important issue in the applications of magnetosomes is the efficiency of biogenic particles in comparison with synthetic magnetic nanoparticles. In [230], the functional applicability of genetically engineered magnetosomes was evaluated and compared with that of commercial immunomagnetic beads. The engineered magnetosomes were fused to protein A and then bonded to antibodies. A previously constructed recombinant MTB strain, *Magnetospirillum gryphiswaldense* ΔF-FA, appeared capable of forming an engineered BMP with protein A on its surface. It has been demonstrated that magnetosomes are characterized by ordered arrangements of bonded antibodies on the surface with fused protein A with a linkage rate of 962 μg Ab per mg of magnetosomes [230]. The complex was used for the detection of *V. parahaemolyticus* surface antigen and hapten, whereas the maximal capture rate was 90% and detection sensitivity was 5 CFU·mL^−1^. Thus, a new engineered BMP fused with protein A (∆F-BMP-FA), coupled with an antibody demonstrated a higher capacity for adsorption of antigen and gentamicin as compared with those of commercial immunomagnetic beads. It has been shown that such particles are inexpensive, eco-friendly, and show a strong potential of applicability as alternatives to commercial immunomagnetic beads, having high Ab-conjugation and antigen-adsorption capacity [230].

A number of authors note that magnetosomes are perspective also as mediators for magnetic fluid hyperthermia and as contrast agents for magnetic resonance imaging, both in vitro and in vivo. Using magnetosomes produced by the magnetotactic bacteria *Magnetospirillum gryphiswaldence* authors of [231] demonstrated that the phospholipid membrane of magnetosomes provides good protection against oxidation and particles are stable over a period of several months. The temperature kinetic relationships obtained for magnetosomes dispersed in an agarose gel under an alternate magnetic field of 17 kA·m^−1^ at 183 kHz frequency demonstrated a rate of temperature rise of 1 °C·min^−1^, which corresponds to a high specific absorption rate (SAR) of 482.7 ± 50.8 W·g^−1^ per mass of iron. Further in [231] the MRI contrast efficiency was also evaluated by means of the acquisition of NMRD profiles for magnetosomes dispersed in agarose gel and in water, showing good results as a negative MRI contrast agent. The MRI experiments on an animal model were carried out with the human glioblastoma–astrocytoma (U87MG) cells inoculated into mice and their presence was detected by magnetic resonance images two weeks after the injection of magnetosomes into the tumor mass thus proving the diagnostic potential of this approach. The high values of relaxivity r2 and the r2/r1 ratio presented in the article [231] show that magnetosomes are efficient superparamagnetic contrast agents for MRI. Further progress in increasing MRI sensitivity and contrast is achieved in [215], where authors developed genetically engineered magnetosomes showing an extremely high relaxivity value of 599.74 mM^−1^·s^−1^. The magnetosomes were extracted from *Magnetospirillum gryphiswaldense* MSR-1 and genetically engineered protein structures of anti-HER2 with the ability to target HER2 were conjugated to the surface layer of the magnetosomes via the anchor protein MamC. This allowed the magnetosomes to target tumors in vitro and in vivo. The magnetosomes did not cause any notable pathogenic effect in the animals, which will greatly advance the development of biogenic magnetic nanoparticles for noninvasive cancer imaging [232].

An effect of increased transverse relaxivity r2 in biogenic MNPs was also noted in earlier works [233]. Studies were carried out with magnetosomes isolated from *Magnetovibrio blakemorei* strain MV-1 and *Magnetospirillum magneticum* AMB-1 which were compared with commercial ferumoxide. The dispersions were studied in vitro and in vivo. Thus, relaxometry measurements at 17.2 T and 20 °C were carried out with phantoms containing agar. The estimated transverse relaxivities r2 for ferumoxide, cuboctahedral magnetosomes from *Magnetospirillum magneticum* AMB-1, and elongated-prismatic magnetosomes from *Magnetovibrio blakemorei* MV-1 were 17.3 ± 15 mM^−1^·s^−1^, 489 ± 26 mM^−1^·s^−1^, 728 ± 35 mM^−1^·s^−1^, correspondingly. Aqueous dispersions were tested in the mouse model and the gain in sensitivity by T2*-weighted imaging at 17.2 T of the mouse brain vasculature was observed after injection of magnetosomes at low concentrations of iron (20 μmol iron kg^−1^). Commercial ferumoxide with the same level of iron did not allow such a phenomenon to be observed [233].

**Figure 18:**
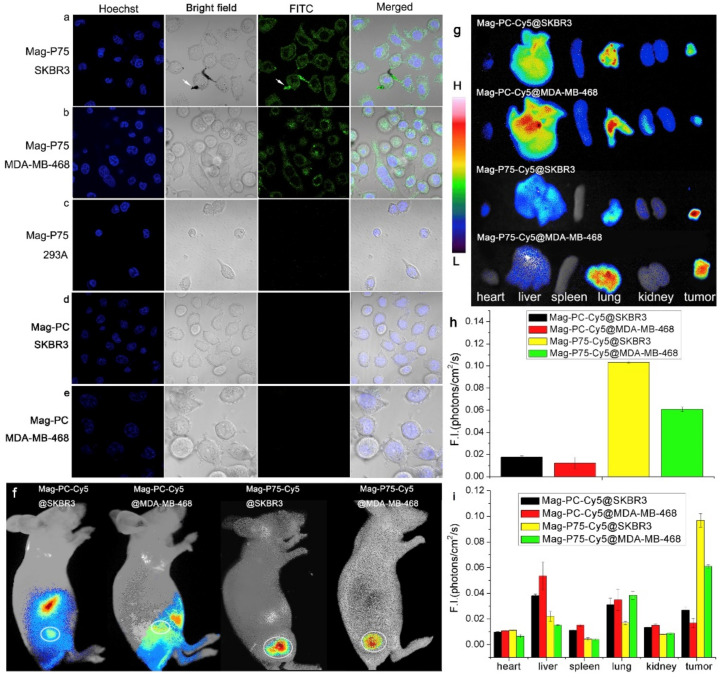
Validation of targeting ability and specificity of Mag-P75 in vivo and in vitro by fluorescence imaging. Confocal analysis of Mag-P75 (labeled with FITC, green) NPs in SKBR3 (**a**), MDA-MB-468 (**b**), and 293A (**c**) cell lines, and Mag-PC (labeled with FITC, green) NPs in SKBR3 (**d**) and MDA-MB-468 (**e**) cell lines, the white arrows indicate the targeting peptides were successfully coupled onto magnetosomes; (**f**) in vivo fluorescence imaging of Mag-PC and Mag-P75 NPs to SKBR3 and MDA-MB-468 tumor-bearing mice; (**g**) ex vivo fluorescence imaging of Mag-PC and Mag-P75 NPs accumulation in tumors and normal organs, and (**h**,**i**) quantification of the fluorescence signals of tumors in vivo and tumors vs. normal organs ex vivo, respectively. Fluorescence intensity was measured in terms of counts/energy/area and is presented with the average value (*n* = 3). Reprinted from [234] with permission of Elsevier provided by Copyright Clearance Center.

An important issue in theranostic approaches on the basis of biogenic magnetic nanoparticles is the modification of the surface. It is noted that such particles are easier to modify due to the specific properties of their surface. In [234], the authors proposed a peptide, for modification of magnetosome surface, showing complementarity to human epithelial growth factor receptor (EGFR) with nanomolar affinity and to epithelial growth factor receptor-2 (HER2) with a lower affinity but comparable to other reported peptides [234]. EGFR is known for being overexpressed in many human epithelial cancers, and thus, could serve as a target for cancer diagnosis and therapy. Authors developed the peptide by screening a computational-aided design of one-bead-one-compound (OBOC) peptide library followed by in situ single-bead sequencing microarray. Two peptides, P75 and P19, were selected to be used as probes for breast cancer cell imaging. The specificity of peptides was tested by confocal fluorescence imaging of the culture with FITC-labeled peptides, incubated for 20 min, and washed. Co-localization analysis was also performed for which AlexaFluor555 conjugated anti-human EGFR antibody was used (Figure 18). Magnetosomes were isolated from *Magnetospirillum gryphiswaldense* (MSR-1) and coupled to targeting peptide P75 by the one-step condensation reaction of amino and carboxyl groups. The transmission electron microscopy (TEM) images of the intact bacteria, magnetosomes, and Mag-P75 with fluorophore indicated that peptide P75 functionalized magnetosomes are well dispersed and have a narrow size distribution. The modified-with-P-75 magnetosome nanoparticles were used for targeted magnetic resonance imaging on the mouse model. The results demonstrated the potential of this peptide for EGFR and HER2-positive tumor theranostics [234] (Figure 18).

#### 3.2.2. Drug Delivery in Cancer Theranostics Using Magnetosomes

The application of nanoscale vesicles for drug delivery in biomedicine has accelerated in recent years and they are now extensively used in patient treatment [235,236]. Magnetosomes isolated from magnetotactic bacteria can be used as carriers of anticancer drugs embedded in their membranes. In [237], the authors bound cytosine arabinoside (Ara-C), in order to reduce its toxic effect, on magnetosome membrane through crosslinking stimulated by the natural biological agent—genipin (GP). The magnetosomes were isolated from *Magnetospirillum magneticum* AMB-1. The complex showed a strongly enhanced controlled drug release effect relieving thus the severe side effects of the drug.

**Figure 19:**
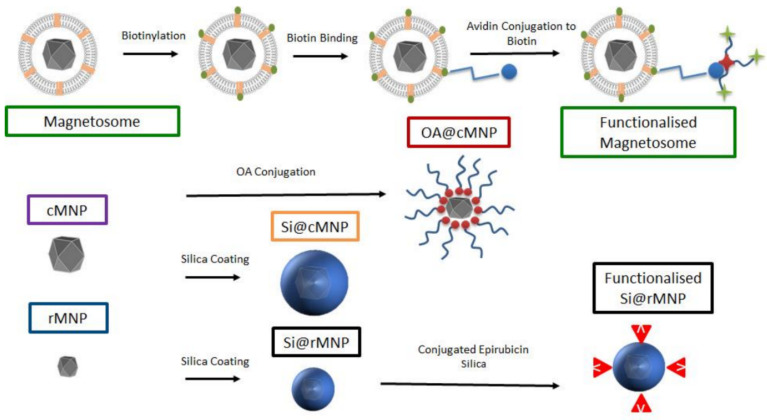
A schematic of the experimental design and samples produced. The top labeled in green depicts the magnetosome and how it is biotinylated for functionalization. Center-left shows the control cMNP labeled in purple with surface coatings of oleic acid (red) and silica (orange label). The bottom-left shows smaller control rMNP (blue label) coated with silica (black label) and with conjugated epirubincin (bottom right). Sample color-code used throughout in figures. Reprinted from [239], license CC BY 4.0.

There are many methods of synthesizing MNPs of different sizes and coating, which can influence their performance as drug delivery vesicles, but magnetosomes are naturally occurring, lipid-coated MNPs that exhibit good stability, homogeneity, biocompatibility, etc. However, their properties, such as cancer cell uptake, toxicity, etc., need further studies. In [238], magnetosomes, synthetic MNP of different sizes, and coated biomimetic MNP were studied for their uptake by MDA-MB-231 cells (estrogen, progesterone, and Her-2 receptor-negative cell line, which serves as a good model of late-stage triple-negative breast cancers). The magnetosome mimics are MNP coated with the oleic acid (OA@MNP) and with silica (Si@MNP) (Figure 19), the latter are of two sizes.

The experiments demonstrated the uptake of particles by MDA-MB-231 cells through inclusion bodies and those particles were located intracellularly. The authors consider that due to the size of the particles, intracellular uptake most likely occurred via pinocytosis with the inclusion bodies being pinosomes or lysosomes, although other processes of MNP internalization including clathrin-mediated endocytosis, etc., are also possible [239]. The particle size was shown to have a negligible effect on overall iron uptake by the MDA-MB-231 cell line. The observed effects of internalization offer the ability to deliver therapeutic compounds directly into the cell and the use of their magnetism to steer the MNP within the body [238].

**Figure 20:**
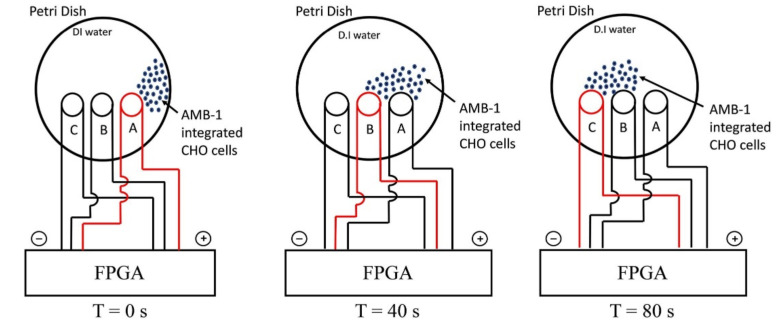
Schematic illustrating the experimental setup for directional control displaying the movement of AMB-1-integrated CHO cells from coil A to coil C. The charged coil is shown in red, and the AMB-1-integrated CHO cells are displayed as blue dots. Reprinted from [240], license CC BY 4.0.

**Figure 21:**
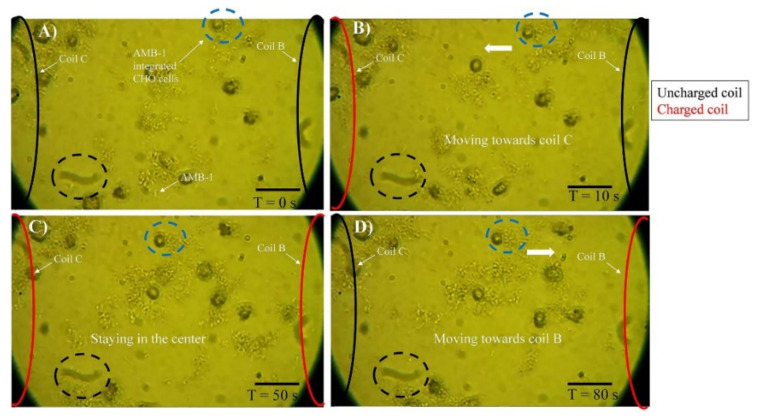
Motility of AMB-1-integrated CHO cells with time. (**A**) At T = 0 s, AMB-1-integrated CHO cells are moving randomly. (**B**) At T = 10 s, coil C on the left is charged and an AMB-1-integrated CHO cell, highlighted by a blue dotted circle, starts to navigate toward the charged coil C. (**C**) At T = 50 s, coils B (right) and C (left) are charged and the AMB-1-integrated CHO cell in the blue dotted circle stays in the center. (**D**) At T = 80 s, only coil C (left) is discharged and the AMB-1-integrated CHO cells can be seen moving toward coil B to the right. A stationary black dotted circle is added at the bottom of every frame to serve as a reference point. The reversal was achieved in the same manner (scale bar = 50 μm). Reprinted from [240], license CC BY 4.0.

The magnetic properties of magnetosomes significantly improve the targeting potential for drug delivery in the presence of a magnetic field. Although the application of such processes obviously demands high levels of monodispersity and reproducibility of size and physical-chemical properties of magnetosomes which in turn ensures a reliable and consistent magnetic response and precise positioning at target tumors. In [240], the controlled navigation capabilities of *Magnetospirillum magneticum* strain AMB-1 (AMB-1) in a magnetic field to target a group of mammalian cells using an in vitro monolayer of Chinese hamster ovary (CHO) cells, including both healthy and tumor cells, was implemented and studied. The motility of MTB cells was controlled by a locally generated magnetic field using ~3-mm-sized solenoid coils forming a network of tracks (Figure 20). At initial time, AMB-1 cells interact with the neighboring CHO cells. When the next coil is charged CHO cells integrated with AMB-1 bacteria are observed moving towards the charged coil (marked with the dashed circle in Figure 20). The direction of the cells’ movement is reversed by switching the order of the coils in the other direction. The authors also studied the interaction of AMB-1 and CHO via computer simulation, by selecting the surface proteins MSP-1 and flagellin of AMB-1 and about 14 potential candidates for CHO. The authors concluded that the mammalian cell surface proteins, which are predominantly responsible for cell signaling, are the primary targets of the AMB-1 cells that are dissuaded by flagellin of AMB-1 (Figure 21). On similar lines, the plasma membrane proteins, whose primary function is to maintain the mammalian cell structure and function, are the targets of the AMB-1 cell surface protein, MSP-1.

Figure 21 presents images obtained with a phase contrast microscope at a magnification of 40×, showing experiments with controlled movement of AMB-1 cells, integrated with CHO cells in a magnetic field. The direction of magnetic field lines is defined by switching the particular coil (C, B). At time T = 0 s, AMB-1 cells exhibit Brownian motion and interact with the neighboring CHO cells. When the coil B is charged at T = 10 s, the CHO cells integrated with AMB-1 bacteria move toward coil B (marked with a dashed blue circle), when coils C and B are charged the cell complex does not move, and when coil B is charged, and magnetic field changes polarity to the opposite, the complex of cells moved in the reverse direction [240].

**Figure 22:**
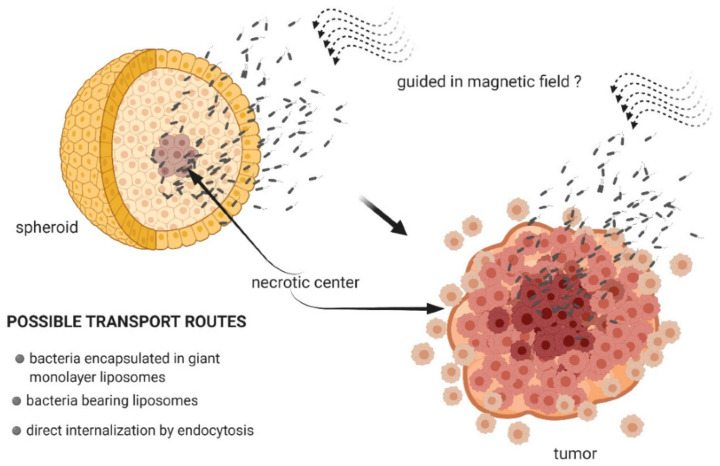
Magnetotactic bacteria as potential drug carriers capable of penetrating the tumor. Reprinted from [242], license CC BY 4.0.

The investigations in magnetosome applications in drug delivery for the treatment of oncological diseases are aimed mainly at achieving the chemical stability of preparations after administration as well as their precise delivery to target tumors and their nearest environment, keeping healthy tissues intact [241]. Biogenic magnetic particles—magnetosomes, perform this task better, due to the stability of their surface layer and their ability to bind specific medications and provide their gradual release. Furthermore, by exploiting their natural magnetotaxis they can be controlled with an externally generated magnetic field. This gives a prospect of guiding magnetosomes in the human body towards the target locations. [241]. Thus, by altering the magnetic field, as shown in [240], it may be possible to control the drug delivery process and move them to the tumor. Some authors [242,243,244] within the framework of this concept call these bacteria specialized nanorobots (Figure 22).

**Figure 23:**
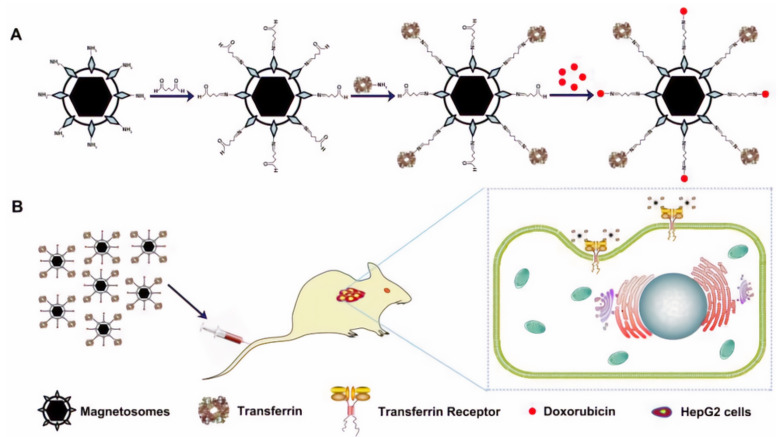
Schematic depiction of formulation of Tf-BMs-DOX (**A**). The antitumor of Tf-BMs-DOX in vivo (**B**). Reprinted from [245], license CC BY 4.0.

In [245], a method for addressing targeting antitumor preparation using magnetosomes was developed on the basis of *Magnetospirillum gryphiswaldense* strain MSR-1, loaded with doxorubicin (DOX) and transferrin (Tf) towards human hepatoma cell line HepG2 and human normal hepatic cell line HL-7702. The simultaneous loading of DOX and Tf on the magnetosomes (Tf-BMs-DOX) enabled an address delivery and binding of complex to the target tumor cells via transferrin receptors (TfR), which are represented on the tumor cells in concentrations of about 100 times higher than in normal cells (Figure 23). The comparative studies with cancer and normal cells demonstrated that the complex Tf-BMs-DOX recognized HepG2 cells more specifically in comparison with HL-7702 because of the high expression of TfR on the surface of HepG2 cells. Data on drug release showed that magnetosomes loaded with DOX were capable of sustained drug release. This means that the frequency of administration and doses could be reduced and the therapeutic effect enhanced. Furthermore, it was observed that the complex Tf-BMs-DOX shows increased tumor cytotoxicity than free DOX or BMs-DOX. The tumor suppression rate was 56.78%, while in free DOX—31.26%. The results obtained in [245] show that magnetosomes modified with DOX and Tf are able to actively target the tumor via intravenous injection (Figure 23).

## 4. Comparative Analysis of the Relevance of Synthesized and Biogenic Particles in Biosensors and Drug Delivery for Cancer Theranostics

**Figure 24:**
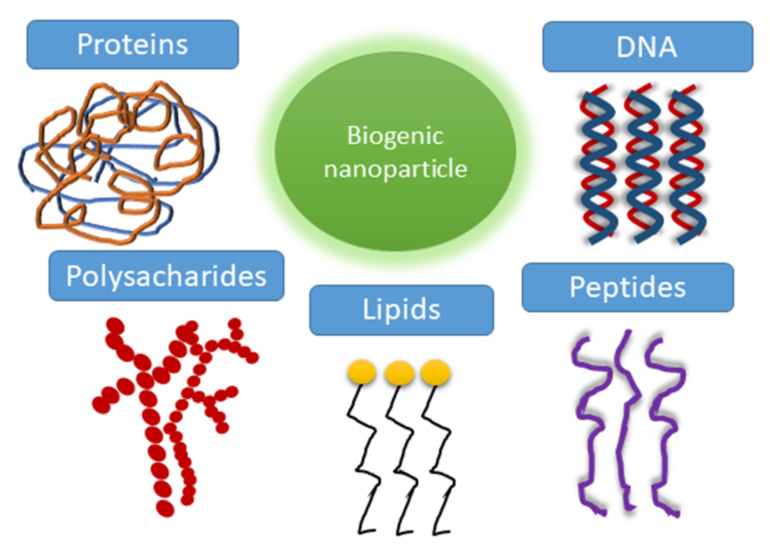
Classes of macromolecules involved in thermodynamic stabilization of biogenic nanoparticles.

Biogenic nanoparticles (BNPs) have been evaluated in a number of studies as eco-friendly and a cost-effective alternative to the chemical synthesis processes [245,246]. The authors of [245] suggest that the advantages of biogenic nanoparticles are due to the natural thermodynamic stability of an organic layer surrounding the magnetic core in these structures. The stability of the structure of biogenic particles could be explained by the presence of different biological macromolecules, such as proteins, lipids, DNA, and polysaccharides, as well as low molecular weight metabolites, such as flavonoids, terpenoids, glycosides, organic acids, and alkaloids—all naturally produced by organisms [246]. Generally, all nanostructures are thermodynamically not stable due to high values of specific surface areas and energy, which leads to the necessity to stabilize them via adding components providing electrostatic, steric, dielectric, etc., stabilizing effects on the dispersion of nanoparticles. The diversity of biological molecules enables various stabilizing effects to be implemented in the nanosystems, including electrostatic repulsion, steric hindrance, van der Waals interaction, etc., which lead to a high degree of stability [246] (Figure 24).

Thus, a key feature of bacterial magnetosomes is the presence of a biological membrane with a defined biochemical composition [247]. This particular coating ensures high quality and homogeneity of dispersions and provides thermodynamic advantages for surface modification [247]. The modification of the surface layer is possible either chemically, or genetically. The second approach provides many advantages since it enables to implement a number of functions at the stage of magnetosome biomineralization [247].

Biosensors are mostly used for in vitro analysis, for which the toxicity issue is not of the primary importance, as that for the drug delivery procedures in vivo. (Although the direction of biosensors for in vivo monitoring begins to develop, it has not yet become a well-established approach [248].) The important features of magnetic nanoparticles for biosensors are: spatial order and stability of the surface layer, capability of chemical modification and bonding of ligands, uniformity of size, homogeneity of magnetic properties, high magnetic relaxivity value [215,231,233,249], and some others. The stability of the organic surface layer in biogenic particles is higher and the structure of the layer is more ordered, respectively the uniformity of the ligands layer is higher in biogenic particles, as noted by a number of authors [241,246]. At the same time, there is a vast diversity of chemically synthesized MNPs, such as “core–shell” type structures (Table 1), and a considerable amount of variants of shells are created, which make it possible to detect various cancer cells with high sensitivity, as well as the availability of high yield processes for the preparation of such MNPs which convinces us of the rationality of application of such particles in mass analysis, PoCT systems and such like applications [69,70]. A unique feature of biogenic nanoparticles is the possibility of application of genetic engineering approaches for their chemical modification which makes it possible to design unique analytical protocols [247]. Thus, it is possible to conclude that in biosensoric applications in vitro, magnetic nanoparticles of biological and inorganic origin, demonstrate close performance with some advantages of biogenic particles. At the same time, the area of MNP synthesis and applications developed a vast diversity of organic and inorganic coatings for MNPs, which is of great value and an opportunity to develop analytical methods for mass biomedical monitoring.

Mobility and targeted delivery problems for magnetic nanoparticles are becoming an actual and important direction of research. Since the formulation of the “magic bullet” concept by Paul Erlich [250], this is the closest perspeсtive of its realization [244]. These bacterial microrobots can be remotely controlled using magnetic fields due to their internal chain of iron oxide nanoparticles acting like a compass needle, as well as, which seems to have more perspective—using magnetosomes as unidomain particles. A comparison of three-varying magnetic field sequences generated by three orthogonal pairs of electromagnets able to generate controllable 3D aggregations of MTB gives a prototype of nanorobots for targeted drug delivery [244]. In cancer therapy, the problem of low internalization of medications in tumor cells and the problem of low internalization of anticancer drugs remains very acute. At the same time, many cancer drugs are expensive and not readily available. These problems make the use of nanocarriers an efficient solution, which improves its therapeutic index via elevating tumor cell internalization and reducing the dose of medication [101]. Another problem is the poor selectivity of anticancer drugs and as a result high toxicity. The address delivery could resolve the toxicity issue [101]. At the same time, many problems with synthetic and biogenic nanoparticles are yet to be solved. Interactions of polymers, including proteins, resulting surface charges, geometry, and energy could have some advantages and disadvantages regarding drug delivery applications [247]. Surface charges can cause aggregation, as well as repulsion, as well as increasing or decreasing the adsorption of the drug onto the surfaces depending on the charges of the drug used.

In [251], the authors compared two different nanoparticles: bacterial magnetosome and HSA-coated iron oxide nanoparticles for targeting breast cancer. Both magnetosomes and HSA-coated iron oxide nanoparticles were chemically conjugated to fluorescent-labeled anti-EGFR antibodies. In vivo MR imaging in a mouse breast cancer model shows the effective intratumoral distribution of both nanoparticles in the tumor tissue. Magnetosomes demonstrated higher distribution than HSA-coated iron oxide nanoparticles according to fluorescence microscopy evaluation. According to the results of in vitro and in vivo study results, magnetosomes are promising for targeting and therapy applications of breast cancer cells [251].

## 5. Conclusions

In recent years, studies demonstrated the advantages of using biogenic nanoparticles in cancer therapy, as well as in vivo visualization of tumors and other pathological neoplasms. These advantages are due to low toxicity, high stability, and spatial order of the organic surface layers. An important property of magnetosomes is the ability to modify their biochemical properties by genetic engineering, which makes it possible to implement unique analytical protocols on biosensor platforms. At the same time, as regards the biosensors and various in vitro applications, the analysis of scientific articles shows that synthetic nanoparticles, as well as biogenic ones, are equally perspective, and further developments as regard shape, surface modification, and analytical protocols are actual and important. In a number of application types, such as mass monitoring of the population, or PoCT, the chemically synthesized MNPs are even preferable, due to relatively low production cost, considerably high yield of the manufacturing processes, sufficient control on the particles size and size distribution, as well as magnetic properties and chemical modification of the surface.

## Figures and Tables

**Table 1:** Applications of synthetic magnetic nanoparticles in biosensors for cancer diagnostics.

Detection Principle		Biorecognition Interface	Target	Detection Limit	Refs.
**Electrochemical**	Square wave voltammetry (SWV)	DNA-modified gold-coated magnetic nanoparticles (DNA-Au@MNPs)	DNA methylation for ovarian cancer diagnosis	2 aM	[60]
	SWV	DNA-Au@MNPs	Circulating tumor DNA (ctDNA)	5 fM	[61]
	Differential pulse voltammetry (DPV)	MWCNT/Fe_3_O_4_ modified with anti-PSA antibodies	Prostate-specific antigen (PSA)	0.39 pg·mL^−1^	[62]
	DPV	Apt-GMNPs	Human T-cell acute lymphocytic leukemia cells (CCRF-CEM)	10 cells·mL^−1^	[63]
	Amperometry	Fe_3_O_4_@GO modified with anti-PSA antibodies	PSA and prostate-specific membrane antigen (PSMA)	15 fg·mL^−1^ and 4.8 fg·mL^−1^, respectively	[64]
	Amperometry	Sox/Pt–Fe_3_O_4_@C/GCE	Sarcosine (prostate cancer biomarker)	0.43 μM	[65]
	Electrochemical impedance spectroscopy (EIS)	MBCPE/Fe_3_O_4_-RGO/PANHS/ssDNA	Breast cancer mutation *BRCA1 5382 insC*	2.8 × 10^−19^ mol·L^−1^	[66]
	EIS	MNPs + antibodies	EpCAM, MUC-1, and HER-2	0.5 μg, 1.0 μg and 0.125 μg per 10^6^ cells, respectively	[67]
	Chronoamperometry	γ-Fe_2_O_3_ /Cr^VI^/Amine Oxidase	Polyamine in tumor tissue	2.47 µM	[68]
	Potentiometry	Anti-AFP with the nanogold/MPS–CoFe_2_O_4_ particles	AFP (α-1-fetoprotein)	0.3 ng·mL^−1^	[69]
**Optical**	Surface-enhanced Raman spectroscopy (SERS)	Magnetic nanoparticle–antibody–CEA–antibody–gold nanoparticle–Raman reporter	Carcinoembryonic antigen (CEA)	10^−12^ M	[70]
	SERS	Raman tags-DNA probes modified Fe_3_O_4_@Ag NPs	MicroRNA in cancer cells (HeLa, MCF-7, A549)	0.3 fM	[71]
	SERS	Magnetic molecularly imprinted polymers (MMIPs) with anti-PSA@DTNB@Au nanoparticles	Prostate-specific antigen (PSA)	0.9 pg·mL^−1^	[72]
	Surface plasmon resonance and MPQ cytometry	Magnetite nanoparticles modified by phytolectins (SBA, WGA, ConA)	Epidermoid carcinoma cells	up to 4.2 ± 0.1 pg·cell^−1^ 2.2 ± 0.5 pg·cell^−1^ and 0.45 ± 0.07 pg·cell^−1^, respectively	[73]
	Surface plasmon resonance	Erlotinib conjugated MNP (erlotinib-MNP)	Human lung cancer cells (A549 cells)	5 µg·mL^−1^	[74]
	UV–vis spectrometry	Au nanoparticles/DNA/magnetic beads	Anterior gradient homolog 2 (AGR2)	6.6 pM	[75]
	Fluorescent detection	DNA/dextran/PAA/Fe_3_O_4_ NPs	p53 protein	8 pM	[76]
	Magnetofluoro-immunosensing (MFI) system	Ag/iron oxide NP-decorated graphene	Prostate-cancer-cell-derived exosome	134.32 NPs·mL^−1^	[77]
	Colorimetry	superparamagnetic iron oxide nanoparticles (SPIONs)/NanoZyme/Transferrine	Transferrin receptors in human U87MG glioblastoma cells	50 cells	[78]
	Colorimetry	Nanocomposite MNP and Pt NP in ordered mesoporous carbon	Human epidermal growth factor receptor 2 (HER2)	1.5 ng·mL^−1^	[79]
**Other principles**	Loop-mediated isothermal amplification (LAMP) and lateral flow device (LFD) with magnetometric detection	Biotin-labeled inner primer and digoxigenin-labeled dUTP and gold magnetic nanoparticle (GMNP) as a signal generator	DNA methylation pattern of miR-34a	-	[80]
	Methylation-specific lateral flow assay (MS-LFA) with magnetometric detection	Amplicon recognizing and capture by gold magnetic nanoparticles (GMNPs)	DNA methylation pattern of miR-34a	0.01 pg	[81]
	Magnetic flow cytometry	Magnetic nanoparticles with aptamers	Pancreatic cancer cells	-	[82]
	Magnetoresistance	Fe_3_O_4_ NPs/Ab in InSb-based semiconductor channel	Liver cancer antigen	0.14 pg·mL^−1^	[83]
	Nanoprobe-based nuclear magnetic resonance (NMR) spectroscopy	Core-shell CoFe_2_O_4_@BaTiO_3_ magnetoelectric (ME) nanoparticles (MENs)	Ovarian carcinoma cells Skov3, glioblastoma cells U87-MG, and breast adenocarcinoma cells MCF-7	-	[84]
	Giant magnetoresistance detection	MoS_2_–Fe_3_O_4_-Aptamer	Exosomes derived from human A431 epidermoid carcinoma cells	100 exosomes	[85]

**Table 2:** The diversity of magnetic nanoparticle cores and shells modification agents for targeted drug delivery in oncology.

Anticancer Drug	Type of MNPs	Coating Agents	Target Cell	Refs.
Adriamycin	Fe_3_O_4_	Homogenous gelatin microspheres	Hepatocellular carcinoma (HCC)	[96]
Bufalin	Fe_3_O_4_	Liposomes	4T1 breast cancer cells	[97]
Camptothecin (CPT)	Fe_3_O_4_	Dextran + folate	Prostate cancer cells	[98]
Сisplatin	Fe_3_O_4_	Amphiphilic polymer + near-infrared dye-labeled HER2 affibody	HER2-expressing tumor cells	[99]
Curcumin (Cur)	Fe_3_O_4_	Bovine serum albumin	MCF7 cells	[90]
Cur	ZnFe_2_O_4_	L-cysteine (L-Cys) + oxygen-containing functional groups and nitrogen-rich mesoporous graphite-phase carbon nitride (Ox, N-rich mpg-C_3_N_4_)	Human lung adenocarcinoma A549 cells	[100]
Cur	Fe_3_O_4_	Hyperbranched polyglycerol (HPG) and folic acid (FA)	HeLa cells	[101]
Doxorubicin (DOX)	Fe_3_O_4_	Polyethylene Glycol (PEG) + polyarabic acid	Human breast cancer cell line MDA-MB-231	[102]
DOX	Superparamagnetic iron oxide nanoparticles (SPIONs)	Poly(ethylene glycol)-poly(aspartic acid) [PEG-P(Asp)] copolymer	Colon carcinoma and fibroblast cell lines	[103]
DOX	mesoporous haematite Fe_2_O_3_	-	Human breast cancer, MCF-7	[104]
DOX	CoFe_2_O_4_	Leucine (Leu)	HeLa cells	[105]
DOX	Fe_3_O_4_	Magnetic molybdenum disulfide (mMoS_2_) + Liposomes	Human breast cancer, MCF-7	[106]
DOX	Ag-Fe_3_O_4_	Dextrin + cell-penetrating peptide (Tat)	MCF-7 cells	[107]
DOX and methotrexate	CoFe_2_O_4_@BaTiO_3_	-	Human hepatocellular carcinoma (HepG2) and human malignant melanoma (HT144)	[108]
Erlotinib (ERL)	SPIONs	Poly N-isopropyl acrylamide (PNIPAM) with aptamer AS1411	Prostate cancer cells	[109]
Growth hormone-releasing hormone antagonist of the MIA class (MIA690)	CoFe_2_O_4_@BaTiO_3_	-	Human glioblastoma cells (U-87MG)	[110]
Hydrophobic anticancer agent ASC-J9	Fe_3_O_4_	Silk fibroin + cationic amphiphilic anticancer peptide, G(IIKK)_3_I-NH_2_ (G3)	Colorectal cancer cells HCT 116	[111]
Methotrexate	Fe_3_O_4_	Arginine	MCF-7, 4T1, and HFF-2 cell lines	[90]
Oxaliplatin (OXA), and irinotecan (IRI)	Fe_3_O_4_	Chitosan (CS)	CT-26 cancer cells	[112]
Paclitaxel (PTX)	SPIONs	FA-conjugated Polyethylene glycol (PEG)/ polyethyleneimine (PEI)-SPIONs SPTX-loaded nanoparticles (SPTX@FA@PEG/PEI-SPIONs)	Nasopharyngeal carcinoma	[88]
siRNA	Fe_3_O_4_	Polyethyleneimine (PEI)	B-cell lymphoma-2 (BCL2), Ca9-22 oral cancer cells	[113]
Sorafenib	Fe_3_O_4_	Mesoporous organosilica + MnO_2_ + hyaluronic acid	Human lung adenocarcinoma A549 cells	[114]
Quercetin 5-fluorouracil	SPIONs	Zeolitic imidazolate frameworks (ZIF) + FA	Breast cancer MDA-MB-231 cells	[115]
Quercetin	MnFe_2_O_4_	Mesoporous hydroxyapatite (HA)	Human breast cancer MCF-7 cells	[116]
Ursolic acid (UA)	Fe_3_O_4_	β-cyclodextrin, folate	Human breast cancer MCF-7 cells	[117]
Violacein	Fe_3_O_4_	Polylactic acid	Glioblastoma and melanoma cancer cell lines	[118]
Zidovudine	NiFe_2_O_4_	Poly(vinyl alcohol)/stearic acid with poly(ethylene glycol) PEG	Human SK-BR-3 breast cancer cell lines	[119]
5-fluorouracil (FLU)	Fe_3_O_4_	(3-aminopropyl) triethoxysilane + tryptophan (TRP)	Human breast cancer MCF-7 cells	[120]
FLU	Fe_3_O_4_-Pt	FLU@PEG nanospheres	4T1 cells	[121]

**Table 3:** Characteristics of magnetosomes from phylogenetically and morphologically identified MTB.

Name of Organism	Crystal Composition	Crystal Shape	Magnetosome			Ref.
			Number	Length (nm)	Width (nm)	
***Alphaproteobacteria***						
*Magnetospirillum caucaseum* SO-1.	Fe_3_O_4_	cuboctahedral	~25	40–50	40–50	[157,158,159,160]
*Magnetospirillum gryphiswaldense* MSR-1	Fe_3_O_4_	cuboctahedral	~30	32–45	32–45	[161,162,163]
*Magnetospirillum kuznetsovii* LBB-42	Fe_3_O_4_	cuboctahedral	~25	40–50	40–50	[164]
*Magnetospirillum magneticum* AMB-1	Fe_3_O_4_	cuboctahedral	~20	~45	~40	[165,166,167]
*Magnetospirillum magnetotacticum* MS-1	Fe_3_O_4_	cuboctahedral	~25	40–50	40–50	[167,168,169]
*Magnetospirillum marisnigri* SP-1	Fe_3_O_4_	cuboctahedral	~25	40–50	40–50	[157,170]
*Magnetospirillum moscoviense* BB-1	Fe_3_O_4_	cuboctahedral	~25	40–50	40–50	[157,171]
*Ca.Magneticavibrio boulderlitore* LM-1	Fe_3_O_4_	prismatic	~15	~50	~40	[172,173]
*Magnetovibrio blakemorei* MV-1	Fe_3_O_4_	prismatic	~10	~55	~35	[174,175,176]
*Ca.Terasakiella magnetica* PR-1	Fe_3_O_4_	prismatic	~15	~45	~35	[177]
*Magnetospira* sp. QH-2	Fe_3_O_4_	prismatic	~15	~80	~60	[178]
***Gammaproteobacteria***						
BW-2	Fe_3_O_4_	octahedral	~30	~65	~60	[179,180]
GRS-1	Fe_3_O_4_	octahedral	~300	~65	~55	[181]
FZSR-1	Fe_3_O_4_	prismatic	~20	~80	~55	[182]
FZSR-2	Fe_3_O_4_	prismatic	~20	~80	~55	[182]
NS-1	Fe_3_O_4_	prismatic	~10	~70	~60	[183]
SHHR-1	Fe_3_O_4_	prismatic	~15	~75	~55	[184]
SS-5	Fe_3_O_4_	prismatic	~20	~85	~65	[180,185]
***Magnetococcia***						
*Magnetococcus marinus* MC-1	Fe_3_O_4_	prismatic	~15	~80	~70	[186,187,188]
*Ca*. *Magnetaquicoccus inordinatus* UR-1	Fe_3_O_4_	prismatic	~30	~75	~45	[189]
*Ca. Magnetococcus massalia* MO-1	Fe_3_O_4_	cuboctahedral	~20	~65	~55	[190,191]
*Magnetofaba australis* IT-1	Fe_3_O_4_	cuboctahedral	~10	~85	~75	[192,193]
***Thermodesulfobacteriota***						
*Ca.Belliniella magnetica* LBB04	Fe_3_O_4_	bullet	~35	~100	~35	[194,195]
*Desulfamplus magnetovallimortis* BW-1	Fe_3_O_4_Fe_3_S_4_	bulletpleomorphic	NDND	~55~33	~35~32	[155,156,196]
*Desulfovibrio magneticus* RS-1	Fe_3_O_4_	irregular/bullet	~10	~40	~20	[197,198,199]
*Ca.Magnetananas rongchenensis* RPA	Fe_3_O_4_	bullet	~70	~115	~40	[200,201]
*Ca*. *Magnetoglobus multicellularis*	Fe_3_S_4_	pleomorphic	60–100	~90	~70	[202,203,204]
***Nitrospirota***						
*Ca*. *Magnetobacterium bavaricum*	Fe_3_O_4_	bullet	~1000	~130	~40	[154,205]
*Ca*. *Magnetobacterium casensis* MYR-1	Fe_3_O_4_	bullet	~1000	~105	~40	[206,207]
*Ca*. Magnetobacterium cryptolimnobacter XYR	Fe_3_O_4_	bullet	~150	~130	~30	[208]
*Ca.Magnetomicrobium cryptolimnococcus* XYC	Fe_3_O_4_	bullet	~100	~135	~45	[208]
*Ca.Magnetominusculus linsii* LBB02	Fe_3_O_4_	bullet	~40	~120	~40	[194,195]
*Ca. Magnetomonas plexicatena* LBB01	Fe_3_O_4_	bullet	~35	~110	~45	[194,195]
***Omnitrophota***						
Ca. *Omnitrophus magneticus* SKK-01	Fe_3_O_4_	bullet	~175	~110	~35	[205,209]

## Data Availability

Not applicable.
